# Phase evolution and morphological transformation of high-entropy alloy FeMnNiAlSiC nanoparticles *via* sequential picosecond laser ablation and nanosecond laser annealing

**DOI:** 10.1039/d5ra03923a

**Published:** 2025-08-11

**Authors:** Bibek Kumar Singh, Yagnesh Shadangi, Harsh Jain, R. Sai Prasad Goud, N. K. Mukhopadhyay, A. P. Pathak, Venugopal Rao Soma, Archana Tiwari, A. Tripathi

**Affiliations:** a Department of Physics, School of Physical Sciences, Sikkim University 6th mile Samdur 737102 Sikkim India ajay_t_2000@yahoo.com; b Department of Materials Science and Metallurgical Engineering, Indian Institute of Technology Bhilai, Jevra-Sirsa Road Durg Chhattisgarh 491001 India; c Department of Ceramic Engineering, Indian Institute of Technology (BHU) Varanasi–221005 Uttar Pradesh India; d Centre for Advanced Studies in Electronics Science and Technology (CASEST), School of Physics, University of Hyderabad Hyderabad 500046 Telangana India; e Centre for Nanotechnology, University of Hyderabad 500046 Telangana India; f Department of Metallurgical Engineering, Indian Institute of Technology (BHU) Varanasi–221005 Uttar Pradesh India; g School of Physics, University of Hyderabad Hyderabad 500046 Telangana India; h DRDO Industry Academia – Centre of Excellence (DIA-CoE;formerly ACRHEM), University of Hyderabad Hyderabad 500046 Telangana India; i Department of Physics, Institute of Science, Banaras Hindu University Varanasi 221005 Uttar Pradesh India

## Abstract

This study investigates the morphological evolution and enhanced crystallinity of FeMnNiAlSiC high-entropy alloy (HEA) nanoparticles (NPs) synthesized using a picosecond laser operating in burst mode and subsequently processed with a nanosecond laser in deionized water (DW). The initial synthesis *via* pulsed laser ablation in liquid (PLAL) revealed distinct phases, like B2, γ-brass, Fe_5_Si_3_, and body-centered cubic (BCC), as confirmed by high-resolution transmission electron microscopy (HRTEM), selected area electron diffraction (SAED), and X-ray diffraction (XRD) data. Elemental mapping indicated enrichment of B2-type phases (Al–Fe and Al–Ni) in the larger NPs, while smaller NPs exhibited γ-brass and Fe_5_Si_3_-type phases. Following nanosecond laser processing, the NPs displayed significant morphological transformations, including the emergence of hollow structures, as well as enhanced crystallinity. Post-processing analysis demonstrated the evolution of B2 and Fe_5_Si_3_-type phases, driven by a laser-induced annealing effect, which resembles the traditional furnace annealing. This dual-stage laser approach effectively combines the rapid synthesis of NPs with structural refinement, offering a versatile pathway for tailoring material properties. These findings underscore the potential of laser-based techniques in the controlled synthesis and structural modulation of HEA NPs, paving the way for applications in catalysis, energy conversion, and advanced functional materials.

## Abstract 2

This study demonstrates that dual-stage laser processing enables the fabrication of phase-controlled, morphology engineered HEA nanoparticles featuring hollow and core–shell architectures.

## Graphical abstract

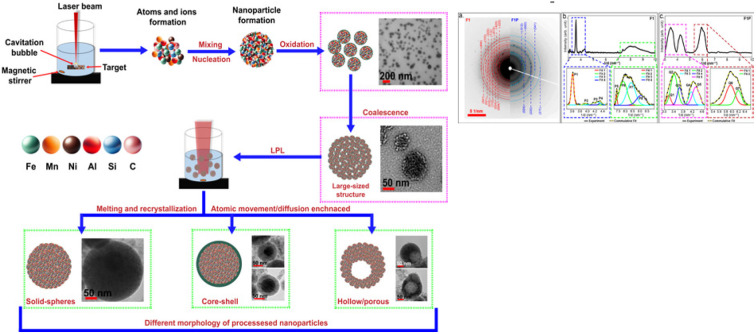

## 1 Introduction

High-entropy alloys (HEAs) represent a revolutionary material class that deviates significantly from traditional alloy design principles. Unlike conventional alloys, which typically consist of one principal element with minor additions, HEAs are composed of multiple elements in near-equiatomic proportions.^1,2^ The concept was introduced in the early 2000's.^3,4^ The exceptional properties of HEAs arise from their complex composition and unique microstructures. These materials often exhibit superior mechanical properties, such as high strength, excellent ductility, and remarkable resistance to wear and corrosion.^5–9^ The multi-element composition results in high configurational entropy, improving stability and performance under elevated temperatures.^8,9^ The versatility in tailoring their composition makes HEAs a promising candidate for a wide range of applications, from aerospace and automotive industries to energy and biomedical sectors.^10–13^

The unique properties of the HEAs make the study of their nanoparticles (NPs) highly desirable. The high surface area, enhanced reactivity, and quantum size effects, in combination with the inherent properties of HEAs opens up new avenues for applications such as energy storage, catalysis, and biomedine.^14–18^ Yu *et al.*^16^ prepared PtPdFeCoNi HEA NPs *via* a high-temperature injection method and demonstrated exceptional catalytic activity and stability in oxygen reduction reaction (ORR), surpassing the performance of conventional Pt/C catalysts. Ai *et al.*^18^ fabricated ultra-small HEA NPs of PtPdRuRhIr *via* a universal ligand cross-linking strategy. Through *in vivo* and *in vitro* experiments, they demonstrated that due to the peroxidase-like activity and photothermal action, the as-synthesized ultra-small HEA NPs can ablate cancer cells and treat tumors. Wang *et al.*^19^ prepared a nanocrystalline CoCrFeNiMn HEA using a laser-inert gas condensation technique, which exhibited high ferromagnetic behavior. They attributed this behavior to the formation of FeCo and NiCo precipitates during the synthesis process and further showed the control over the saturation magnetization and Curie temperature of the alloy through heat treatment. In a separate study, Wang *et al.*^20^ reported the enhancement in the hardness of nanocrystalline CoCrFeNiMn HEA after annealing, which was maintained upto 1100 °C. This was attributed to the combination of small grains and Cr-rich phases present in the HEA NPs. Broge *et al.*^21^ studied the formation of HEA NPs composed of PtIrPdRhRu using a solvothermal synthesis method at 200 °C. They reported the formation of HEA NPs through an autocatalytic mechanism, where initial Pd nuclei catalyze the reduction of other metal ions, resulting in homogeneous alloy NPs exhibiting FCC structure. The synthesis required a precise temperature and extended reaction time. Exceptional mechanical properties of HEA across a wide range of temperature were showcased by Yang *et al.*^22^ by leveraging a dual-high entropy strategy. By combining multicomponent matrix with NPs, the strength and ductility both were enhanced, with a yield strength of approximately 1 GPa and an ultimate tensile strength of 1.45 GPa and a significant elongation of 46% at room temperature. Wu *et al.*^23^ synthesized HEA NPs composed of platinum-group metals, using a wet chemical method resulting in FCC structured NPs, and studied its catalytic performance. They observed a superior catalytic activity of these NPs in ethanol oxidation reaction (EOR) in comparison to the commercially available catalysts such as Pd/C, Pd black, and Pt/C. The higher catalytic activity was attributed to the diverse adsorption sites and high stability of the HEA NPs. Yang *et al.*^24^ explored the aerosol synthesis of HEA NPs, by nebulizing an aqueous solution of metal salts into aerosol droplets, which are then rapidly heated and quenched. They showed HEA NPs with atomic level mixing of otherwise immiscible metal elements, and further emphasized the advantage of this method in producing HEA NPs with uniform elemental distribution. Tahir *et al.*^25^ explored how different target preparation methods can affect the morphology, composition, and crystallinity of HEA NPs synthesized *via* pulsed laser ablation in liquid (PLAL). They discovered that powder-pressed heat-treated target produced solid solution HEA NPs with highest productivity and a near-equiatomic composition that closely matched the target. In contrast, targets prepared *via* ball-milling resulted in NPs with a core–shell structure when subjected to PLAL, their study highlighted the significant influence of the target preparation method on the properties and productivity of HEA NPs.

There are several methods available for the synthesis of HEA NPs, such as high-temperature injection method, wet chemical methods, solvothermal synthesis method, aerosol synthesis method, mechanical ball milling, carbothermal shock synthesis method, sol–gel auto combustion method, plasma-arc discharge, PLAL *etc.*^2,16,21,23,24,26–31^ Out of the above-mentioned synthesis techniques, PLAL provides advantages over other synthesis routes in terms of faster synthesis time, purity of NPs, control over sizes and shapes. In addition, it's a one-step synthesis process capable of generating NPs of metals, semiconductors, and HEAs as well.^32–37^ The synthesis of HEA NPs using PLAL has been demonstrated in,^15,29^ and the effect of laser processing in liquid (LPL) has been demonstrated in our previous study.^2^ Watanabe *et al.*^38^ reported the synthesis of FeCoNi medium entropy alloy NPs using a high repetition-rate UV picosecond LAL. Their results showed the formation of Ni-rich FeCoNi-core, Fe-shell NPs and chain-like structures due to the over-striking ablation process. Gatsa *et al.*^39^ introduced a multi-beam PLAL using diffractive optical elements to enhance NPs synthesis efficiency. By bypassing the cavitation bubble shielding, this approach significantly increased the production of CrFeCoNiMn HEA NPs, offering a cost-effective alternative for industrial applications. Fieser *et al.*^14^ synthesized CuCoMn_1.75_NiFe_0.25_ HEA NPs using femtosecond laser ablation in various liquid media, achieving high production rates and near-stoichiometric compositions. They observed core–shell morphologies, oxygen vacancies, and laser-induced NPs chaining *via* nonthermal mechanisms. Their study emphasized electrocatalytic performance, particularly in aluminium–air batteries, demonstrating enhanced ORR, HER, and OER activity. When working with HEA materials, it has to be understood that the microstructural stability of HEAs is strongly dependent on the temperature.^8,40^ The HEAs are often subjected to annealing to induce structural changes, morphological changes, and phase transformations.^40–42^ The traditional annealing typically performed in a furnace is time-consuming. To overcome this, Li *et al.*^4^ adopted a CW laser instead to realize the annealing effect and induce phase transitions. They reported that by varying the irradiation time, NPs with different crystalline structures can be obtained. As the irradiation time increases, more crystalline phases were observed to evolve. This was attributed to the slower cooling rates at longer irradiation times. In addition, burst-mode laser ablation has emerged as a transformative technique in materials processing, leveraging the rapid delivery of multiple ultra-short pulses to enhance ablation efficiency and precision.^43,44^ Compared to single-pulse ablation, burst modes allow cumulative energy deposition, improved thermal management, and higher throughput, making them ideal for applications requiring controlled surface structuring and efficient material removal.^45,46^ Moreover, the interplay of successive pulses with plasma and material dynamics in burst modes significantly influences ablation outcomes, offering unique advantages in tailoring NPs synthesis.^47^ However, while extensive studies have focused on the energy efficiency and surface quality of burst-mode ablation, comparatively less attention has been directed toward the characteristics of the ablated material itself, particularly the NPs formed during these processes.

In the present study, a picosecond laser operating in burst mode (10 pulses per burst) was used to generate the HEA NPs in deionized water (DW) *via* PLAL. Subsequently, these NPs were subjected to laser processing in liquid (LPL) using a nanosecond laser with an 8 ns pulse duration. This dual-stage laser treatment led to significant morphological changes, notably the formation of hollow HEA NPs, alongside solid spherical and core–shell structures. DW was chosen as the ablation medium to maintain a clean environment for NPs formation, ensuring that no external species interfered with elemental distribution or phase evolution during synthesis and processing. High-resolution transmission electron microscopy (HRTEM), selected area electron diffraction (SAED), and X-ray diffraction (XRD) analyses revealed the presence of B2, γ-brass, Fe_5_Si_3_-type, and body-centered cubic (BCC) phases in the ablated NPs, with enhanced crystallinity and the evolution of specific phases post-processing. The observed Fe_5_Si_3_-type phases indicate that the laser processing induces an annealing effect similar to traditional furnace annealing. This study highlights the potential of pulsed laser techniques to tailor the structural and morphological properties of HEA NPs, paving the way for their application in advanced functional materials.

## 2 Materials and methods

### 2.1 Target preparation

High-purity elemental powders of Fe, Ni, Mn, Al, Si, and C (99.2% purity) were weighed in appropriate stoichiometric ratios to achieve the desired Fe_40_Mn_19_Ni_15_Al_15_Si_10_C (at%) composition. The powder mixture was subjected to mechanical alloying using a planetary ball mill (Retsch PM 400/2) operating at 200 rpm with a ball-to-powder weight ratio of 10 : 1. Milling was conducted in tungsten carbide (WC) vials using WC balls, with toluene as the process control agent to prevent oxidation during milling. After 35 hours of continuous milling, the resulting alloyed powder was compacted into a cylindrical pellet of 3 mm thickness and 1 cm diameter, which was subsequently used as the target material for pulsed laser ablation in liquid (PLAL) experiments. The structural properties of the pellet are reported in ref. 48.

### 2.2 Laser ablation and processing

The Fe_40_Mn_19_Ni_15_Al_15_Si_10_C HEA target was subjected to PLAL using a picosecond laser (INNOSLAB FXz50-3-GF), operating in burst mode (10 pulses per burst), at a laser wavelength of 1030 nm, pulse duration of 4 ps, repetition rate of 1 kHz, and laser energy of 270 μJ. DW was used as the solvent for the ablation (liquid height above the target was 8 mm), and the ablation time was fixed at 55 minutes. During the ablation process, the target was moved using a translation stage at 50 μm sec^−1^ speed. The beam diameter before focusing was 3 mm, and after focusing, the beam spot size was measured to be ∼56 μm. The number of bursts fired between two spots was ∼700. The colloidal solution obtained post ablation, is labelled as F1, was further subjected to LPL for 15 minutes using a nanosecond laser (Litton Laser, LPY 707 G-10), operating at a laser wavelength of 1064 nm, pulse duration of 8 ns, repetition rate of 10 Hz and laser energy of 60 mJ. For the processing, the laser beam was focused at the centre of the liquid, having a focused spot area of 1.5 × 10^−3^ cm^2^. The processed colloidal solution is labelled as F1P.

### 2.3 Characterization

The NPs obtained after ablation (F1) and processing (F1P) were subjected to morphological and structural analysis. Transmission electron microscopy (TEM) micrographs, High-resolution TEM (HRTEM) micrographs, elemental mapping of the synthesized NPs, and selected area electron diffraction (SAED) patterns were obtained by JEOL: JEM f200, operating at 200 keV. The TEM micrographs are a representation of the batch population. Gatan microscopy software was used to obtain the particle size distribution, fast-Fourier transform (FFT), and inverse fast-Fourier transform (IFFT) images. Radial profiles from SAED patterns were obtained using imageJ software. To extract the radial profile, the “Radial Profile” plugin was used, which calculates the average pixel intensity as a function of radial distance from the center. The plugin averages intensity values along concentric circles, effectively converting the 2D ring pattern into a 1D plot of intensity *versus* radius. This transformation provides a quantitative representation of diffraction ring intensity, allowing for the identification of distinct phases based on peak positions. The X-ray diffraction (XRD) pattern of the bulk target before annealing was obtained with EMPYREAN, PANALYTICAL equipped with a Co-k*α* source (*λ* = 1.7902 Å). The XRD of the bulk target after annealing, and the NPs were recorded using PAN-ALYTICAL Spectris Technologies, PW3040/60, equipped with a Cu-k*α* source (*λ* = 1.5406 Å).

## 3 Results and discussion

### 3.1 Morphological and structural characterization of F1 and F1P

#### 3.1.1 Morphological characterization

**Fig. 1:**
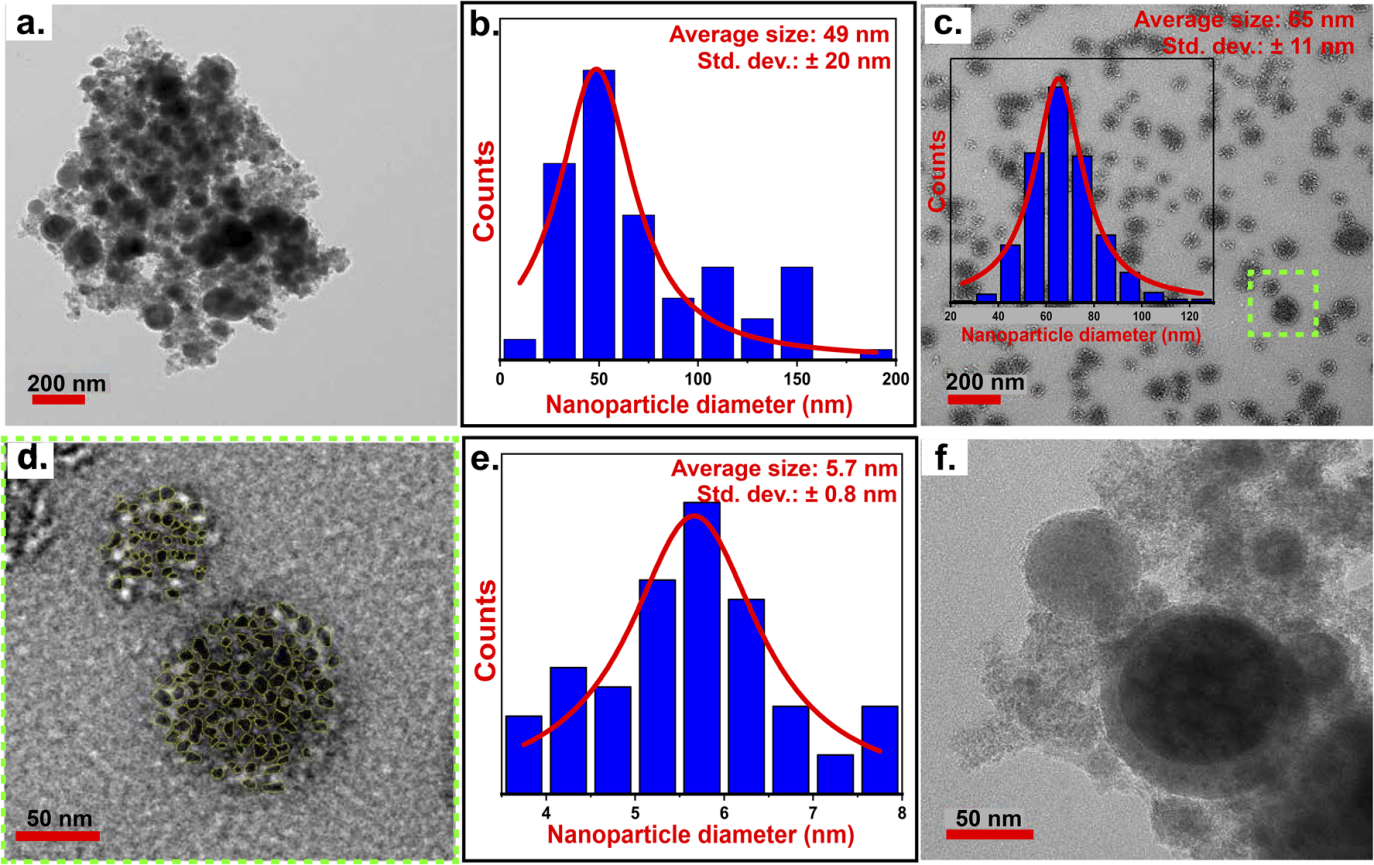
(a) TEM micrographs of NPs showing spherical morphology; (b) particle size distribution of particles observed in (a); (c) TEM micrograph of large-sized structures formed by the coalescence of small-sized NPs with its size distribution in the inset; (d) zoomed region of (c) marked with green box; (e) particle size distribution of particles observed in (d); (f) core–shell NPs observed in F1.

The NPs (F1) obtained after the ablation of Fe_40_Mn_19_Ni_15_Al_15_Si_10_C_1_ showed spherical shape with an average size of 49 nm (standard deviation (*σ*) = ± 20 nm) (Fig. 1(a and b)). In addition, smaller NPs were also observed. However, these tiny particles tended to coalesce together, forming large-sized structures with an average size of 65 nm (*σ* = ± 11 nm) (Fig. 1(c)). These large-sized structures were made up of smaller NPs that ranged in size from 3 to 8 nm, with an average of 5.7 nm (*σ* = ±0.8 nm) (Fig. 1(d and e)). The coalescence of small-sized particles is due to the presence of Fe in high concentration on the surface of the NPs, which gets oxidized due to the DW medium. The magnetic nature of Fe-oxides acts as a bridge to facilitate the coalescence of the particles,^49^ and form large-sized structures (Fig. 1(c)). A close inspection of these large-sized structures (Fig. 1(d)), revealed its porous nature, which is attributed to the gap/void generated during the coalescence of the particles arising due to their size mismatch. In addition, solid spherical NPs and core–shell structured NPs were also observed (Fig. 1(f)). Quantitative analysis of the F1 sample showed 88.16% of the particles were solid spherical NPs, while 11.84% exhibited a core–shell morphology (Fig. 3). The limited visibility/presence of the core–shell structures may be due to the formation of thin shells with low contrast. The oxidation of the NPs was confirmed by absorbance spectra (discussed in Section 3.2.1). The solvent environment strongly influences the formation of core–shell structures during laser ablation. Core–shell structures observed in the NPs synthesized in DW are attributed to oxidation-induced elemental segregation. Elemental mapping (Fig. 6) shows that Al and Fe are more concentrated in the shell region in comparison to the other constituent elements. The presence of Al at the edges is due to its low melting point (Table 1). The use of DW, an oxygen-rich solvent, facilitates the formation of surface oxides during ablation, promoting outward diffusion of these elements. This aligns with observations by Rawat *et al.*,^50^ where DW resulted in core–shell structures, while ethanol favored solid solutions due to its lower oxidative potential. Interestingly, Fieser *et al.*^14^ reported oxide-related UV features in ethanol under prolonged ablation, suggesting that even low-oxidation media can support shell formation over time.

**Table 1:** Physical and chemical properties of elements present in HEA sample^2,63,64^

Elements	Atomic radius (Å)	Melting point (K)	Crystal structure	Valence electron concentration (VEC)
Fe	1.241	1811	BCC	8
Al	1.438	933.3	FCC	3
Mn	1.40	1519	Complex cubic	7
Si	1.18	1687	Diamond	4
Ni	1.246	1728	FCC	10
C	0.773	3823	FCC	4

**Fig. 2:**
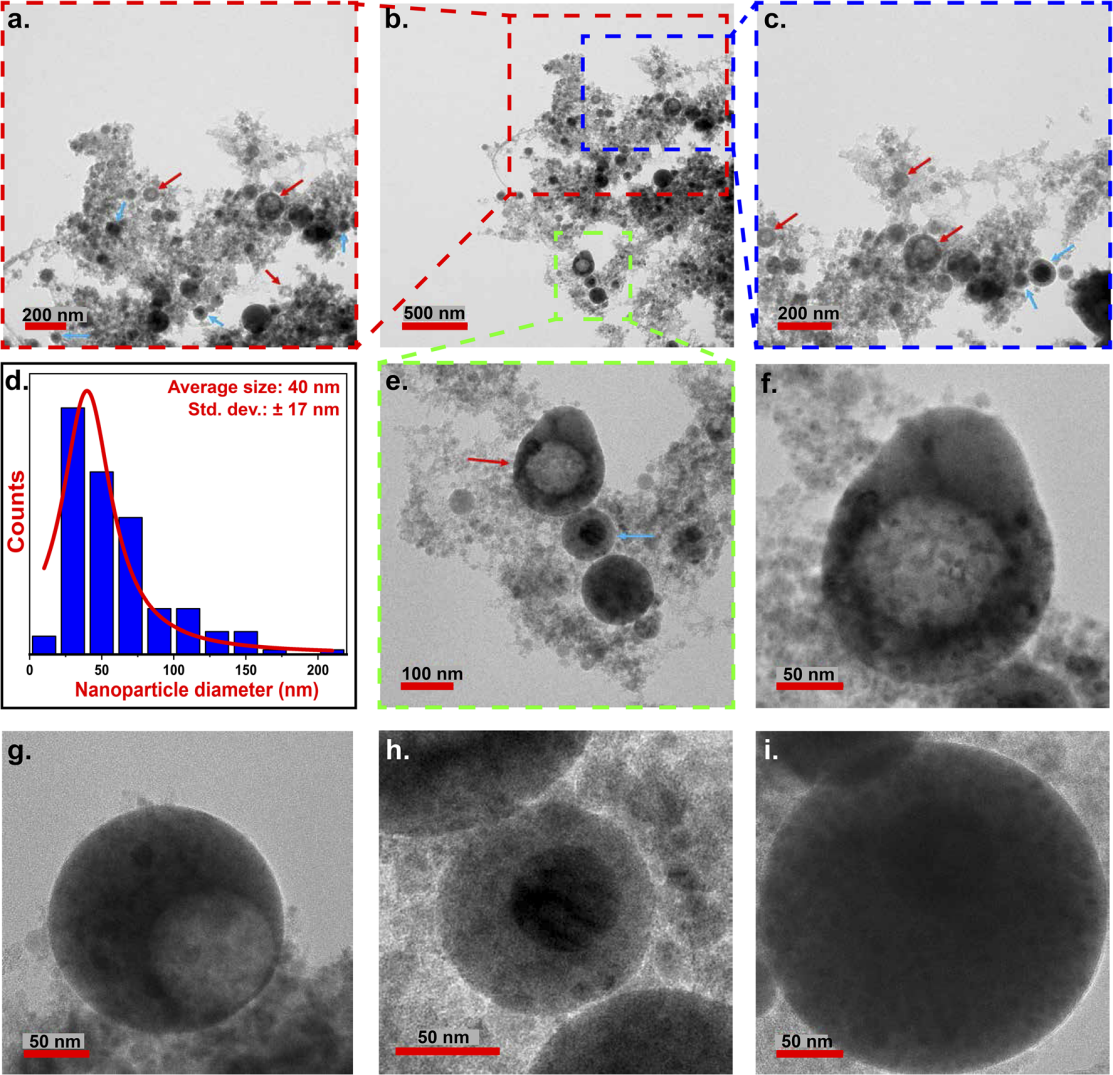
(a–c) TEM micrographs of NPs (red arrows indicates the hollow NPs and the blue arrows indicates the core–shell NPs), (d) particle size distribution of particles observed in (b) and (e) zoomed image of the region marked in (b) with dashed green box, (f and g) hollow NPs (h) core–shell NPs, (i) solid spherical NPs observed in F1P.

**Fig. 3:**
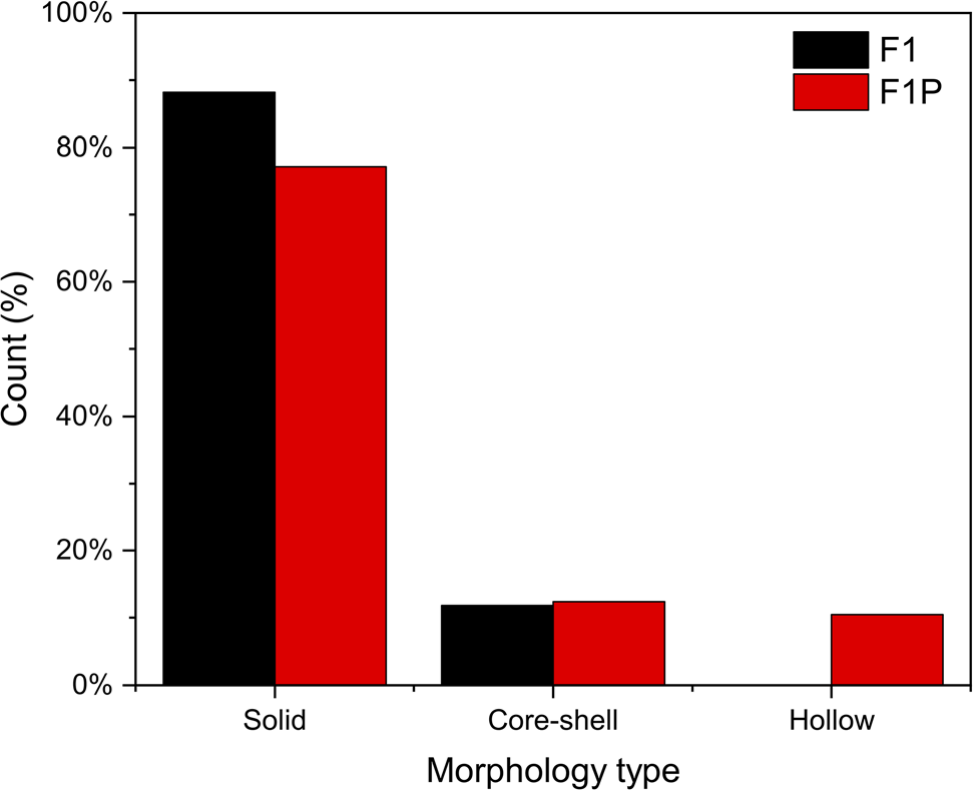
Quantification of solid spheres, core–shell, and hollow NPs in F1 (sample size (*n*) = 76) and F1P (*n* = 105).

When F1 was later subjected to LPL with a nanosecond pulsed laser, significant changes in their morphologies were observed. The TEM images obtained from the processed sample (F1P) is shown in Fig. 2. The F1P NPs showed spherical shape with an average size of 40 nm (*σ* = ± 17 nm) (Fig. 2(d)). Post-processing, in F1P showed the absence of the large-sized structures that were observed in F1. In F1P, in addition to the solid NPs, presence of core–shell structures (indicated with blue arrows in Fig. 2(a, c and e)), as well as hollow NPs (indicated with red arrows in Fig. 2(a, c and e)) were also observed. Fig. 2(f–i), shows NPs showing the different morphologies mentioned above, namely, the hollow NPs (Fig. 2(f and g)), core–shell structure (Fig. 2(h)), and solid NPs (Fig. 2(i)). Morphological quantification of the F1P sample indicated 77.14% of the particles were solid NPs, 12.38% were core–shell structures, and 10.48% were hollow (Fig. 3). The formation of different morphologies of the NPs, can be explained by the following processes occurring simultaneously during the laser processing:

(1) The large-sized structures undergo fragmentation upon the laser irradiation,^2,33^ resulting in the generation of small-sized NPs. In F1P, a dominant group of particles with an average size of 17 nm (±2 nm) was also observed (shown in Fig. S1(a) in the SI). These small particles upon re-irradiation can again merge together *via* the melting and resolidification process,^2,33^ resulting in the formation of solid NPs.

(2) The large-sized structures can also directly undergo melting, without being fragmented,^33^ and eliminate the voids during the resolidification.

(3) The high temperature state of the order of 10^3^ K, attained during the laser-processing, can cause asymetric Ostwald ripening,^51,52^ resulting in the formation of hollow NPs. In this process, the voids of the large-sized structures merges and grows into a larger void, pushing the remaining particles towards the edges of the NP.^51^

(4) Further, oxidation can lead to the formation of core–shell structures.

**Fig. 4:**
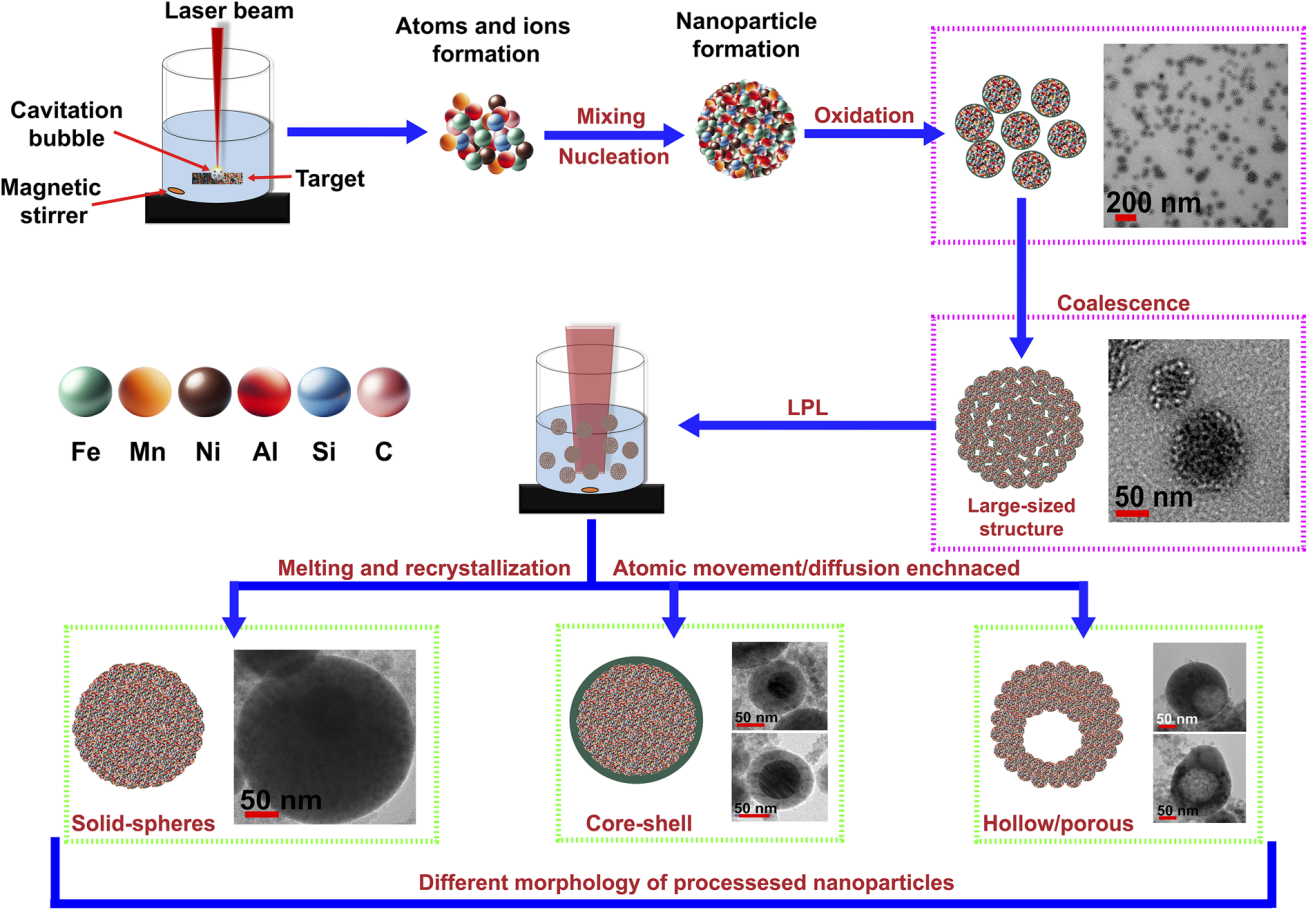
Schematic showing the mechanism for the growth of different morphologies of the NPs observed in F1 and F1P.

Fig. 4 provides a schematic diagram, summarizing the growth mechanisms and morphological evolution of the NPs in both F1 and F1P.

#### 3.1.2 Structural characterization

**Fig. 5:**
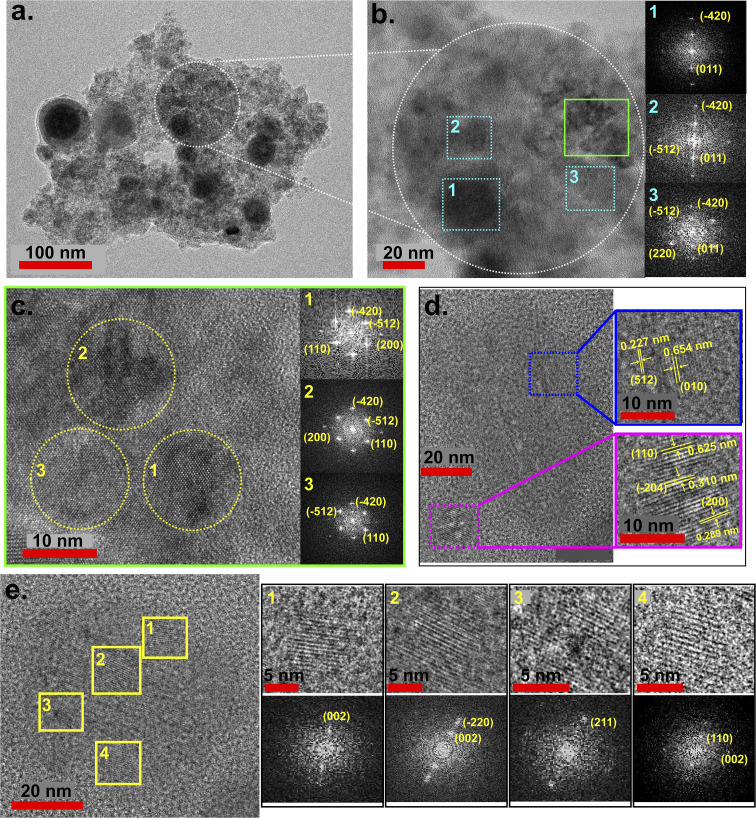
(a) TEM micrograph of F1 NPs, (b) zoomed image of the region marked in (a) (white dotted-circle), along with the FFT images taken from three different regions marked as 1, 2, and 3 (blue font), (c) zoomed image of the region marked in (b) (green box), along with the FFT images taken from three different regions marked as 1, 2 and 3 (yellow font), (d) HRTEM images of two small NPs along with zoomed lattice fringes, (e) HRTEM of a NP with the lattice fringes zoomed from four different regions marked with yellow box and labeled as 1, 2, 3 and 4 along their corresponding FFT images.

Fig. 5 shows the HRTEM images and the corresponding FFT images obtained from various NPs of F1. From the FFT of region 1, 2, and 3, marked in Fig. 5(b), *d*-spacing values corresponding to the B2-type phase (0.284 nm and 0.272 nm corresponding to (−512) and (−420) planes, respectively), Al–Fe–Ni type phase (0.503 nm corresponding to (011) plane), and γ-brass type phase (0.314 nm corresponding to (220) plane) were obtained. The enlarged view of the region marked with green box in Fig. 5(b), is shown in Fig. 5(c), where again three different regions were considered. From here, we obtained the *d*-spacing values corresponding to BCC phase (0.183 nm corresponding to (200) plane), B2-type phase (0.284 nm and 0.272 nm corresponding to (−512) and (−420) planes, respectively), and Fe_5_Si_3_ type phase (0.33 nm corresponding to (110) plane). From Fig. 5(d and e), *d*-spacing values of B2-type phase (0.59 nm, 0.358 nm, 0.310 nm and 0.227 nm corresponding to (002), (−220), (−204) and (521) planes, respectively), Al–Fe–Ni type phase (0.654 nm corresponding to (010) plane), γ-brass type phase (0.625 nm and 0.366 nm corresponding to (110) and (211) planes, respectively), and Fe_5_Si_3_ type phase (0.336 nm and 0.289 nm corresponding to (110) and (200) planes, respectively) were obtained [JCPDS No.: 00-029-0042, 01-083-3994, 00-003-1052; 00-040-1132; 01-071-0397; 00-011-0615, 01-074-4749]. The high miscibility of Al with Fe (Δ*H*_mix_ = −11 KJ mol^−1^, Table 2) and Ni (Δ*H*_mix_ = −22 KJ mol^−1^, Table 2), and having the same space-group (*Fm*3̄*m*), allows it to readily form Al–Fe and Al–Ni phases, leading to the observation of B2-type phases.^41,42,53,54^ The B2-type phase is also preferred in HEA, due to the high entropy effect (high configurational entropy), further rapid cooling has also been reported to stabilize the B2-type phases in HEAs.^53–55^ In addition to B2-type phases (Al–Fe and Al–Ni phases), Al–Fe–Ni phases were also observed. Fe and Ni are miscible Δ*H*_mix_ = −2 KJ mol^−1^, Table 2, but they lack the capability of forming phases.^56,57^ However, the presence of Al in the mix, leads to the formation of Al–Fe–Ni phases, as observed. Si despite having high enthalpy of mixing [Δ*H*_mix_ = −35 KJ mol^−1^ (Table 2)] with Fe, has low solubility in Fe matrix,^58,59^ which can restrict Fe–Si phase formation. However, in our study, formation of Fe_5_Si_3_-type phase was observed. This is attributed to the unique dynamics of burst-mode ablation. Unlike single-pulse ablation, burst-mode delivers multiple closely spaced pulses, where subsequent pulses interact with the ablated material, inducing localized heating.^45–47^ This transient heating enhances atomic mobility, similar to an annealing effect, facilitating Fe_5_Si_3_-type phase formation. This is in agreement with the reports of enhancement of mixing of Si in Fe at higher temperatures,^60–62^ and also explains the formation of Fe_5_Si_3_-type phases in the NPs, as observed through the HRTEM analysis. The γ-brass type phases (also present in the bulk target), is observed due to Mn, however, such phases have not been observed to dominate in the NPs. The phases observed in the case of F1 were also present in F1P. Fig. S1(b) in SI, shows the HRTEM images and corresponding phases identified from F1P NPs.

**Table 2:** Enthalpy of mixing (Δ*H*_mix_) of atomic pair for the constituent elements of the HEA target (values taken from ref. 65)

Alloys	Δ*H*_mix_ (KJ mol^−1^)	Alloys	Δ*H*_mix_ (KJ mol^−1^)
FeAl	−11	AlC	−36
FeNi	−2	NiSi	−40
FeMn	0	NiMn	−8
FeSi	−35	NiC	−39
FeC	−50	MnSi	−45
AlNi	−22	MnC	−66
AlSi	−19	SiC	−39
AlMn	−19		

**Fig. 6:**
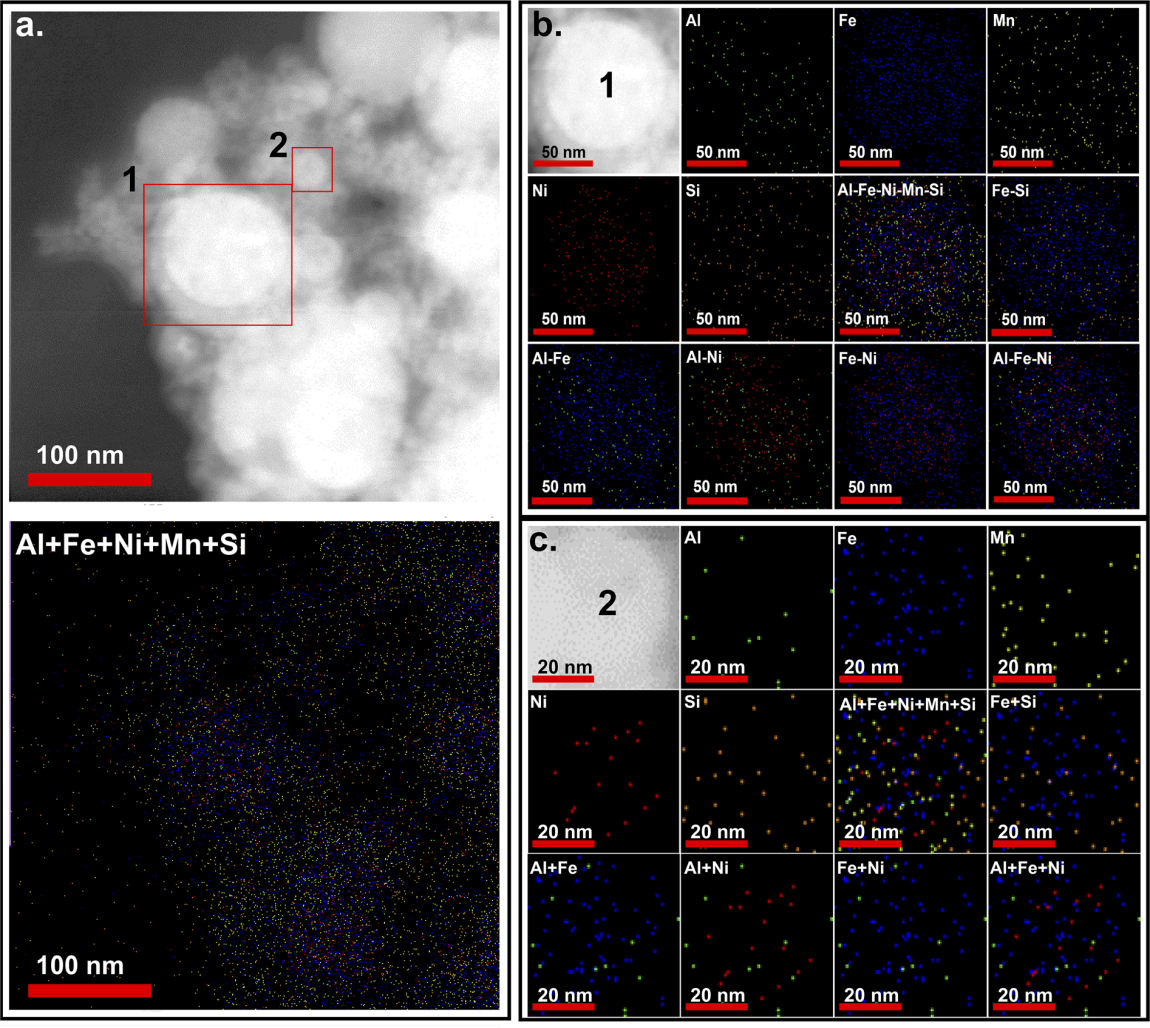
(a) SEM image of F1 NPs and its elemental mapping, (b) elemental mapping of the particle marked as 1 in (a), and (c) elemental mapping of the particle marked as 2 in (a).

The elemental mapping of F1 NPs is shown in Fig. 6(a), which shows a homogeneous distribution of elements. The mapping of carbon (C), is not displayed in the figure. Due to the use of a C-based substrate, the concentration of C was detected to be very high. Considering, its low at% in the target material (1%), and its small size, which allows it to dissolve in the matrix faster as reported in ref. 48 the focus was on the other elements present in the NPs, assuming that C is distributed homogenously and retained its concentration within the NPs. Additionally, the Raman spectrum (Fig. S2, SI) did not show any carbon-related peaks, indicating that there is no significant increase in carbon content in the synthesized NPs. For completeness, the carbon mapping is also provided in the SI (Fig. S3(a)), which shows a high C content homogeneously distributed; however, this is attributed to the use of a carbon-based grid substrate, as discussed above. The observation supports the assumption that the carbon content is close to the target composition (1 at%). Furthermore, the absence of new phases in the synthesized NPs suggests that there was no significant increase in carbon concentration during ablation. However, the more quantitative techniques such as XPS would be required for definitive confirmation.

**Fig. 7:**
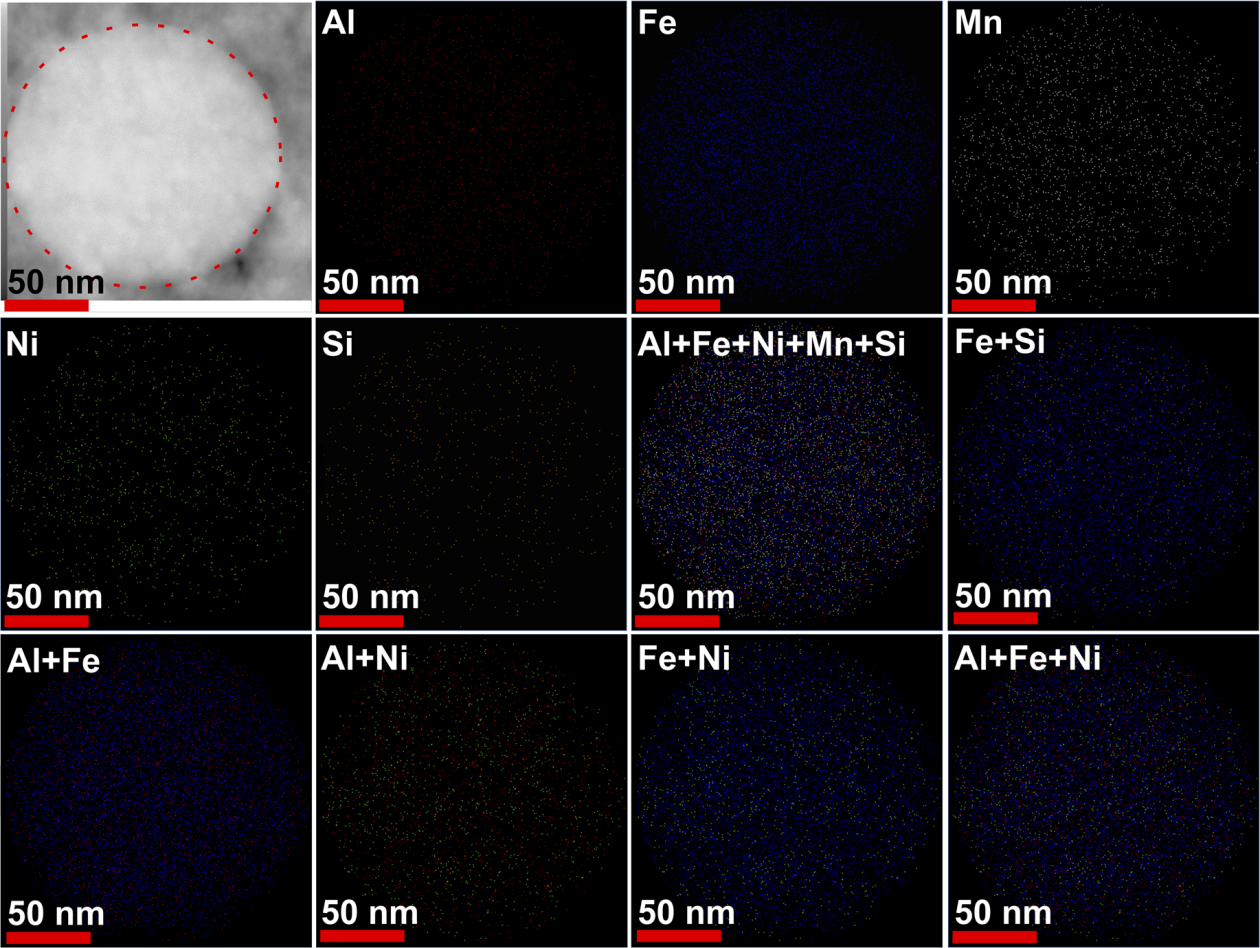
SEM image of a F1P NP and its elemental mapping.

To understand the phase formation in the NPs, two particles marked as 1 and 2 in Fig. 6(a), were analyzed. The elemental mapping of particle 1 and 2 is shown in Fig. 6(b) and (c), respectively. In the larger NP (particle 1, Fig. 6(b)), the edges of the particle consisted of all the elements except Ni, whereas the central region consisted a homogeneous distribution of all the elements. Upon examining the binary mixing map, the formation of Al–Fe, Al–Ni, Fe–Ni, Fe–Si, and Al–Fe–Ni phases seems more likely to form, which is in agreement with phases detected with the phases identified *via* HRTEM analysis. In contrast, the smaller NP (particle 2, Fig. 6(c)) reveals that Si, Mn, Ni, and Fe are more concentrated toward the center, with Al distributed at the edges. This pattern suggests that γ-brass-type and Fe_5_Si_3_-type phases likely dominate in smaller particles. However, in the HRTEM imaging of the smaller-sized particles, the presence of B2-type phases was also noticed. To further investigate the phase distributions, two additional particles were examined, as shown in Fig. S4 in SI. In the larger particle, a distribution trend similar to particle 1 was observed, with Al, Fe, and Ni concentrated at the central region. However, in the smaller particle, Al was also present at the central region, although in lower concentration, with Ni more pronounced toward the edges. Here, Si and Mn remain concentrated at the core alongside Fe. These findings confirm the dominance of B2-type phases in larger particles and suggest a competitive presence of multiple phases in smaller NPs. Specifically, in smaller NPs, a higher concentration of Mn and Si as compared to Al and Ni favors the formation of γ-brass-type and Fe_5_Si_3_-type phases over the B2-type phase. Fig. 7 shows the elemental mapping of a F1P NP. Here all the elements were observed to be homogeneously mixed. Post-processing, a significant enhancement in the uniformity of distribution of all the elements was observed from the mapping images. This shows, during the LPL atomic mobility was promoted, which leads to rearrangement of atoms resulting in a more homogeneous distribution of all the constituent elements. The line scan of F1 and F1P NPs were also obtained (shown in Fig. S6 and S7, respectively, SI), which agrees with the elemental distribution of the NPs discussed above.

From the EDS of F1 (shown in Fig. S8 (a), SI), the concentration of the constituent elements were obtained. Assuming the at% of C in the synthesized NPs to be sustained at 1% (as discused above), the at% of the other constituent elements were re-calculated (Table 3) and was used to calculate the physio-chemical parameters (such as, atomic size deviation(*δ*), and valence electron concentration (VEC)); and thermodynamic parameters (such as, configurational entropy of mixing (Δ*S*_mix_), enthalpy of mixing (Δ*H*_mix_), melting temperature (*T*_m_)) to check the criteria for the formation of solid solution.^2,66–68^ The definition of these parameters is given in the SI file (eqn (4)–(10)). As discussed in the literature, *Ω* = 1 can be regarded as the critical boundary value for the formation of solid solution. When *Ω* > 1, the *T*Δ*S*_mix_ is sufficiently large and favors the stabilization of solid solution, whereas *Ω* < 1, increases the likelihood of the formation of intermetallic phases.^68^ Δ*S*_mix_ = 12.32 J mol^−1^ K^−1^, Δ*H*_mix_ = −37.27 KJ mol^−1^, *δ* = 7.91, VEC = 6.24, and *Ω* = 0.55 were obtained. The obtained VEC value suggests the formation of BCC phases. In addition, the value of *Ω* obtained here indicates the formation of intermetallic phases rather than the solid solution phases. These numbers are consistent/in agreement with the BCC, B2-type (ordered BCC phases), and Fe_5_Si_3_-type intermetallic phases observed in the NPs. Similarly, the physio-chemical and thermodynamic parameters for F1P using the concentration of the constituent elements obtained from the EDS of the sample (Table 4 and Fig. S9(a), SI) of the NPs were also calculated. And Δ*S*_mix_ = 10.43 J mol^−1^ K^−1^, Δ*H*_mix_ = −15.60 KJ mol^−1^, *δ* = 7.14, VEC = 7.05, and *Ω* = 1.1 were obtained. Contrary to the case of F1, the numbers obtained for *Ω* and VEC, indicates the formation of solid solution with a mixture of BCC and FCC phases.^2,66–68^ Indicating a phase transition from high-entropy intermetallic phase to solid solution high entropy alloys (HEAs) post-processing. The transition from pure BCC phase to a mixture of BCC and FCC phase, after LPL has also been observed in a previous study.^2^ This highlights the strength of LPL to induce phase changes and modify the structural properties of HEA NPs. Further, the EDS from a different region of F1P sample consisting smaller-sized NPs was obtained (shown in Fig. S9(b), SI). The thermodynamic parameters were calculated again, yieding Δ*S*_mix_ = 12.32 J mol^−1^ K^−1^, Δ*H*_mix_ = −30.53 KJ mol^−1^, *δ* = 7.91, VEC = 6.49, and *Ω* = 0.66. The value of *Ω* indicates a tendency toward intermetallic phase formation rather than a solid solution. This behavior is attributed to the high concentration of Si and lower concentration of Al, present in the NPs of the region under consideration. With Al being in lower concentration which is a B2-type phase stabilizer, the higher concentration of Si, drives the formation of intermetallic phases primarily in the smaller-sized NPs (also observed in case of F1 NPs, through elemental mapping and HRTEM). Whereas, in case of bigger-sized NPs, where concentration of Al is higher, B2-type phase is dominant and this leads to the formation of solid solution instead of intermetallic phases. To verify this, F1 sample was checked for the concentration of Si and Al (Fig. S8(a and b), SI), and Si was found to be in comparatively higher concentration. This suggests that an increased Si concentration can restrict the formation of solid solution in HEA NPs. For completeness, the mapping for C and the Raman spectra of F1P sample is shown in Fig. S3(b) and S5 respectively in the SI.

**Table 3:** Original and recalculated atomic percentages for F1 sample

Element	Original (%)	Original *σ*	New (%)	New *σ*
C	81.21	0.84	1.00 (fixed)	—
Al	1.52	0.19	8.01	1.00
Si	6.33	0.55	33.37	2.90
Mn	3.13	0.44	16.50	2.32
Fe	6.26	0.65	33.00	3.43
Ni	1.54	0.35	8.12	1.85

**Table 4:** Original and recalculated atomic percentages of F1P sample

Element	Original (%)	Original *σ*	New (%)	New *σ*
C	64.80	0.65	1.00 (fixed)	—
Al	4.93	0.29	13.87	0.82
Si	1.96	0.19	5.51	0.53
Mn	4.96	0.48	13.95	1.35
Fe	20.83	1.02	58.60	2.87
Ni	2.51	0.39	7.06	1.10

#### 3.1.3 Annealing effect in LPL

**Fig. 8:**
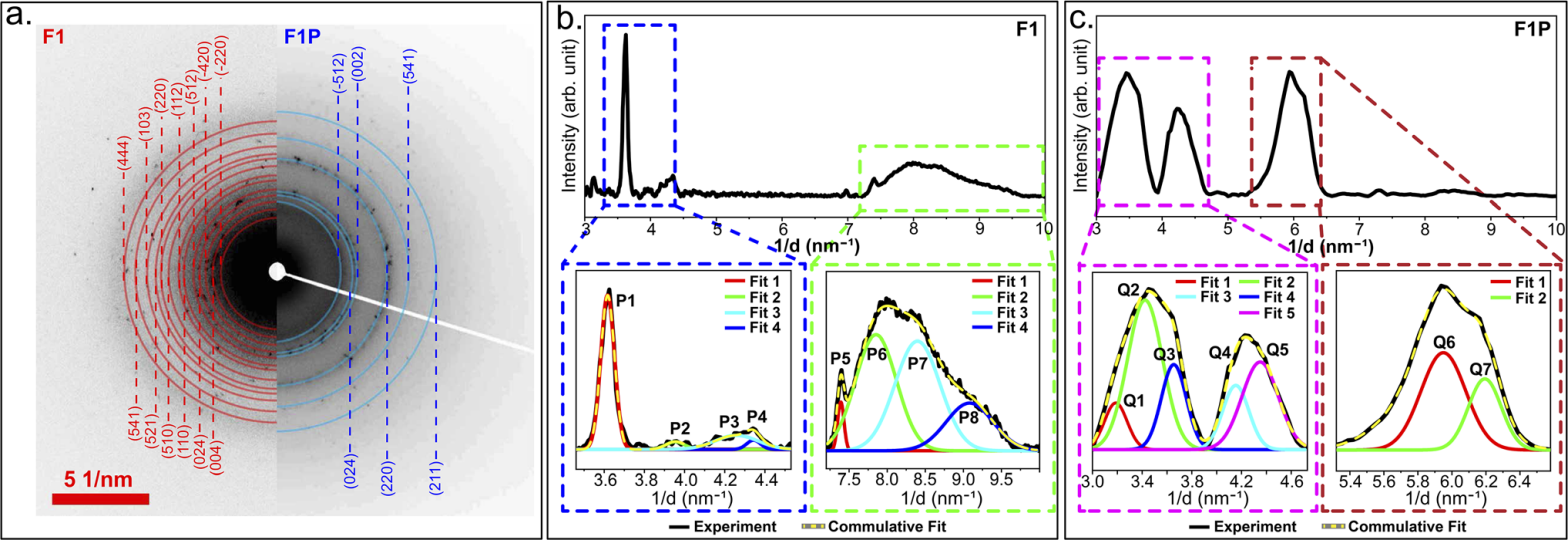
(a) SAED pattern of F1 (left-side) and F1P (right-side); (b) radial profile of SAED pattern of F1 and; (c) radial profile of SAED pattern of F1P.

The SAED pattern obtained for F1 is shown in Fig. 8(a) (left-side). Similar to the HRTEM, the SAED pattern revealed *d*-spacings corresponding to BCC phase, B2-type phase, γ-brass type phase, and Fe_5_Si_3_-type phases (the *d*-spacings and corresponding crystal planes are mentioned in Table 5). The radial profile of the SAED pattern was further analyzed (Fig. 8(b)) and in the inset two prominent zones of the profile are highlighted. The regions shown in the inset were deconvoluted and several peaks were identified. The peaks P1, P3, and P4 corresponds to the B2-type phase; P2, P5, P6, and P7 corresponding to the γ-brass type phase; and, P2 and P8 corresponding to the Fe_5_Si_3_-type phase (Table 5). For comparison, the SAED pattern of F1P is shown on the right-side of Fig. 8(a). Similar to F1, the presence of B2-type phase, BCC phase, γ-brass-type phase, and Fe_5_Si_3_-type phase was also observed here (Table 5), and the corresponding radial profile shows the same (shown in Fig. 8(c), with enlarged view of two prominent regions shown in the inset with deconvoluted peaks).

**Table 5:** Phases identified from the SAED pattern and its corresponding radial profile of F1 and F1P

F1		F1P	
SAED	Radial profile	SAED	Radial profile
0.351 nm (220)^a^	P1 → 0.276 nm (220)^a^	0.281 nm (−512)^a^	Q1 → 0.314 nm (220)^d^
0.298 nm (004)^a^	P2 → 0.253 nm (222)^d^	0.242 nm (024)^a^	Q2 → 0.298 nm (004)^a^
0.276 nm (−420)^a^	P3 → 0.235 nm (002)^c^	0.234 nm (002)^c^	Q3 → 0.274 nm (−420)^a^
0.242 nm (024)^a^	P4 → 0.230 nm (−332)^a^	0.165 nm (220)^c^	Q4 → 0.242 nm (024)^a^
0.227 nm (512)^a^	P5 → 0.135 nm (541)^d^	0.137 nm (541)^d^	Q5 → 0.230 nm (−332)^a^
0.203 nm (110)^b^	P6 → 0.128 nm (444)^d^	0.117 nm (211)^b^	Q6 → 0.167 nm (220)^c^
0.198 nm (−530)^c^	P7 → 0.119 nm (642)^d^	—	Q7 → 0.162 nm (521)^d^
0.191 nm (112)^c^	P8 → 0.110 nm (114)^c^	—	—
0.176 nm (510)^d^	—	—	—
0.169 nm (220)^c^	—	—	—
0.162 nm (521)^d^	—	—	—
0.147 nm (103)^a^	—	—	—
0.137 nm (541)^d^	—	—	—
0.128 nm (444)^d^	—	—	—

a → B2-type phase.

b → BCC phase.

c → Fe_5_Si_3_-type phase.

d → γ-brass type phase [JCPDS No.: 00-029-0042, 01-083-3994; 00-006-0696; 01-071-0397; 00-011-0615,01-074-4749].

The SAED pattern of F1 and F1P were compared. In the SAED pattern of F1 (Fig. 8(a), left-side), many diffraction spots were observed. However, its radial profile (Fig. 8(b)) showed only one strong peak (P1) corresponding to B2-type phase, followed by a broad peak (in the region of 7 nm^−1^ to 9.5 nm^−1^), accommodating four peaks (P5, P6, P7, and P8). The peaks P2, P3, and P4 were observed to be very weak. Moreover, several planes that were observed through SAED pattern were not observed in its radial profile, due to poor contrast of the spots, suggesting its weak presence. In comparison to the diffraction rings observed in the SAED pattern of F1 (Fig. 8(a), left-side), in case of F1P, the diffraction rings are more prominent (Fig. 8(a), right-side). The changes are also evident from its radial profile (Fig. 8(c)). The sharp peak P1, as observed in case of F1, is now replaced by two strong peaks (in the region of 3 nm^−1^ to 4.5 nm^−1^), whose deconvolution resulted in five peaks corresponding to B2-type (Q2, Q3, Q4, and Q5) and γ-brass type phases (Q1) (Table 5). The planes that were identified from the SAED pattern of F1, but were missing in its radial profile or were having weak presence are now clearly detected in F1P, namely Q2 (004), Q4 (024), and Q5 (−332), all associated with B2-type phase (Table 5). Another notable change in F1P is the disappearance of the broad peak observed in the region of 7 nm^−1^ to 9.5 nm^−1^ in F1. Instead, a strong peak appeared in the 5.4 nm^−1^ to 6.4 nm^−1^ range. Deconvolution of this region revealed two peaks, namely, Q6 (220) associated with Fe_5_Si_3_-type, and Q7 (521) associated with γ-brass type phase were observed (Table 5). As compared to F1, Fe_5_Si_3_-type phase became more prominent in F1P. The planes corresponding to Q6 ((220), Fe_5_Si_3_-type phase) and Q7 ((521), γ-brass type phase) were also observed in the SAED pattern of F1 (Fig. 8(a), left-side), however, it was missing from its radial profile. The presence of these missing planes in the radial profile of F1P, suggests the enhancement in formation of these planes (and corresponding phases) in F1P NPs. These observations indicate notable phase evolution and rearrangement of atoms leading to more ordered orientation of crystal planes due to laser-induced annealing. Laser induced annealing has been reported to transform amorphous thin films to a crystalline structure in the literature.^69–72^ Minissale *et al.*,^73^ used laser heating in their study and reported the improvement in microstructure of tungsten after the annealing. The evolution of the phases in F1P can be explained as following-the nanosecond laser pulse delivers energy gradually over an extended duration, thereby slowing down the cooling rate post-pulse as compared to ultra-short pulses.^74^ This allows atoms more time to rearrange and form a highly ordered crystalline lattice during the resolidification process.^74,75^

**Fig. 9:**
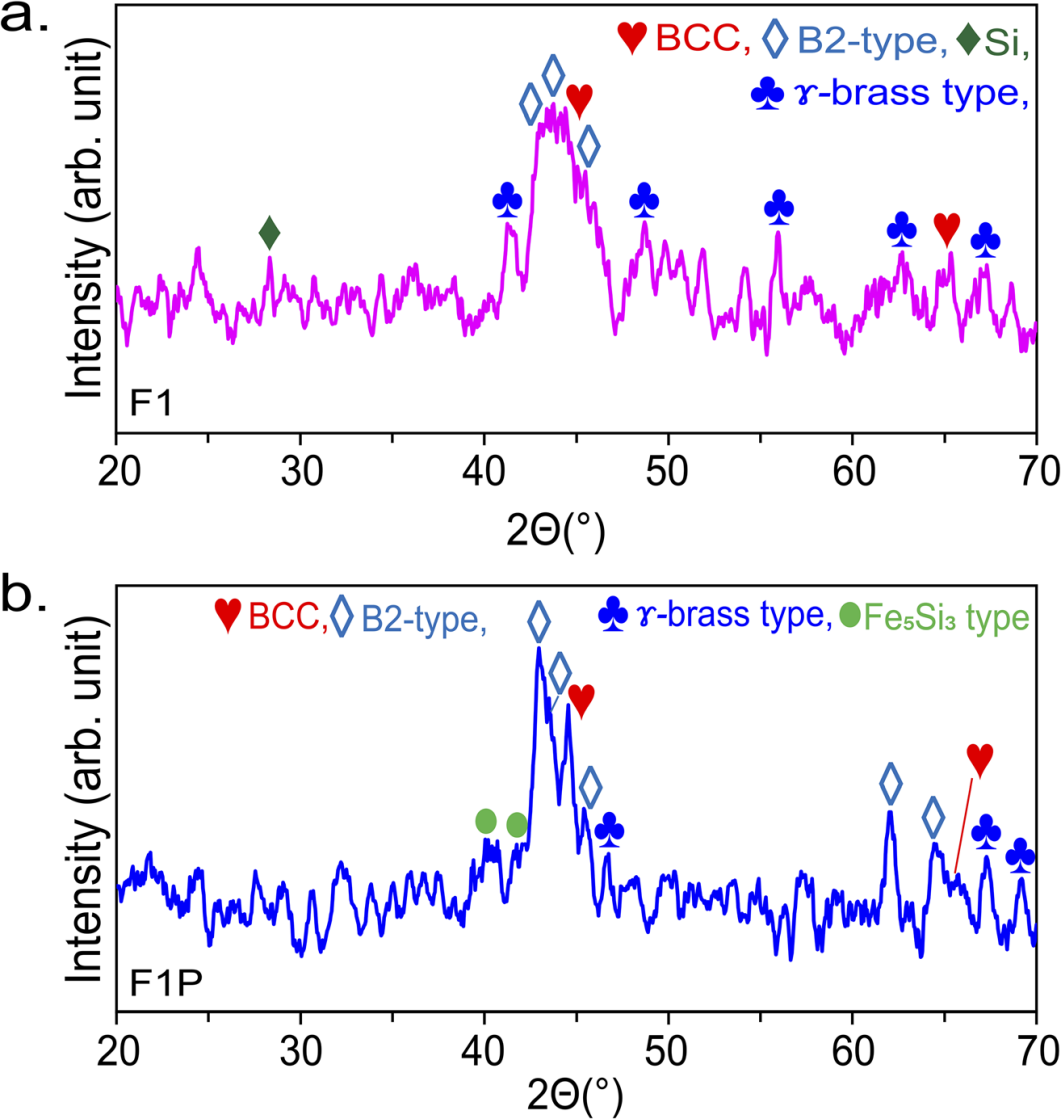
XRD pattern of (a) F1 and (b) F1P.

**Fig. 10:**
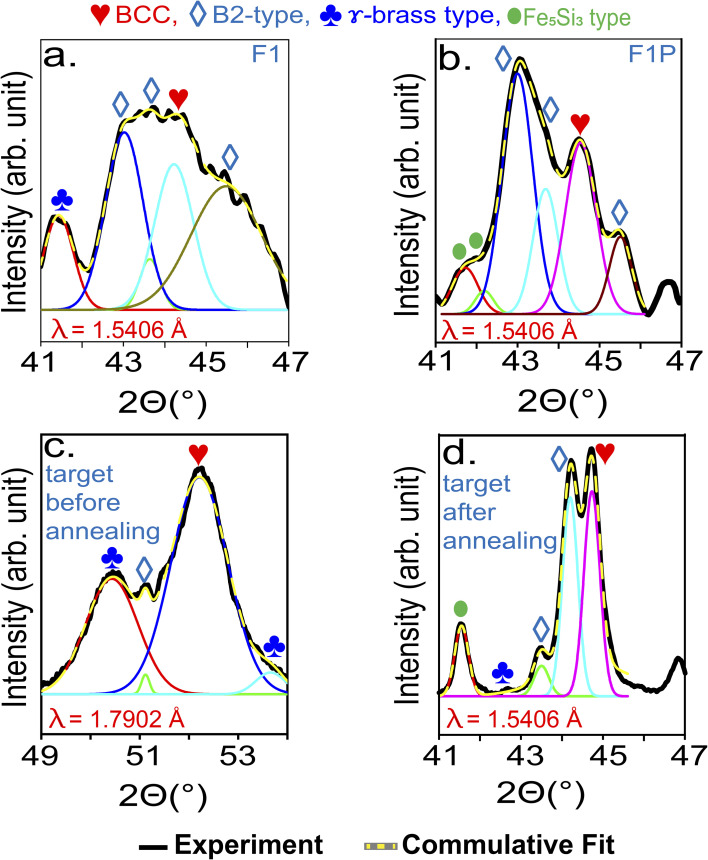
XRD pattern of (a) ablated NPs (F1), (b) processed NPs (F1P), (c) bulk target, and (d) bulk target after annealing (800 °C).^48^ (The XRD pattern shown in (a), (b) and (d) are recorded with Cu-k*α* source, *λ* = 1.5406 Å, and the XRD pattern shown in (d) is recorded with Co-k*α* source, *λ* = 1.7902 Å).

For further clarification, the XRD patterns of the samples were obtained. The XRD pattern of F1 NPs is shown in Fig. 9(a), and the phases identified are listed in Table 6. From the XRD pattern, dominance of B2 is evident. The phases identified in the XRD pattern confirmed the retainment of the targets phase in the NPs (XRD pattern of the target material is shown in Fig. S10, SI). This can be understood as following- during ablation a rapid thermal process is initiated but it is short-lived. The NPs are ejected into gas phase, and due to rapid solidification, the diffusion of atoms is restricted, thereby freezing the structure/atoms position in place which helps in the retainment of the targets structure. At the same time, the fast cooling and heating owing to the burst mode operation of laser, results in the formation of amorphous phases/poor crystalline structure.^74^Fig. 9(b) shows the XRD pattern of F1P NPs, and the phases identified are listed in Table 6. The Si peak observed in F1 (at 28.53°) was not observed in F1P, indicating Si dissolved completely after processing. Comparing the XRD pattern of F1 and F1P, the broad peak observed in F1 (Fig. 9(a)) between the range 41°–47° gets sharper in F1P(Fig. 9(b)), suggesting enhancement in the crystallinity of the NPs after being subjected to nanosecond laser processing. The broadness of the diffraction peaks in the above mentioned region, were checked through deconvolution. The deconvoluted peaks (Fig. 10(a)) of F1, exhibited a full-width half maximum (FWHM) ranging from 0.58° to 2.02°. The broad peak suggests the presence of significant lattice strain and small crystallite domain, indicating partial amorphous nature. In contrast, the deconvoluted peaks in F1P, revealed a general reduction in FWHM (ranging from 0.52° to 0.88°), with a new peak appearing at 2*θ* angle of 42.18°. The FWHM of these deconvoluted peaks decreased significantly as compared to F1 (by a factor of 2). This reduction indicates a substantial increase in crystallite size, attributed to the laser-induced annealing effect.

**Table 6:** Phases detected from XRD pattern of F1 and F1P

F1			F1P		
28.53°	→	Si	40.02°	→	Fe_5_Si_3_
41.43°	→	γ-brass	42.18°	→	Fe_5_Si_3_
43.02°	→	B2	42.99°	→	B2
43.63°	→	B2	43.64°	→	B2
44.22°	→	BCC	44.56°	→	BCC
45.58°	→	B2	45.51°	→	B2
48.47°	→	γ-brass	46.58°	→	γ-brass
56.15°	→	γ-brass	62.02°	→	B2
62.64°	→	γ-brass	64.59°	→	B2
65.1°	→	BCC	65°	→	BCC
67.11°	→	γ-brass	67.28°	→	γ-brass
	—		69.17°	→	γ-brass

For more insights, the XRD patterns of the target before and after annealing are shown in Fig. S10 and S11 in SI, respectively. The phase evolution in the target material due to annealing is studied by Jain *et al.*^48^ In their study, BCC and B2-type phases were identified as the major phase in the target material, followed by γ-brass type. In addition, a Si peak (also observed in case of F1) was noted. After annealing, this peak related to Si was absent, which indicated its dissolution in the alloy system. Post-annealing, Fe_5_Si_3_-type phases were also observed. For comparison with F1 and F1P, prominent region of the XRD pattern of target material before and after annealing is shown in Fig. 10(c) (49° to 54°) and Fig. 10(d) (41° to 47°), respectively. Unlike F1, F1P, and the annealed target, XRD pattern of the target before annealing appears shifted, due to the use of a different X-ray source (Co-k*α* source, *λ* = 1.7902 Å). In the target, B2-type phase is observed to have enhanced after annealing. In conclusion, F1 NPs retained the phases present in the target material. In addition, Fe_5_Si_3_-type phases were also formed, which is attributed to the brief annealing due to the use of burst mode operation of laser for ablation. In F1P, B2-type phase is observed to have enhanced (Fig. 10(b)), similar to the case of annealing of the target material (Fig. 10(d)). Meanwhile, BCC phase became less intense than B2-type phase but remained as one of the major phases. Diffraction peaks related to Fe_5_Si_3_-type phases were also detected more in F1P (Fig. 9(b)), which is again similar to the case of annealing of the target (Fig. S11, SI). These results shows that due to the high temperature involved during the laser processing, phase change/evolution can be induced similar to the case of bulk being subjected to furnace annealing treatment.

### 3.2 Optical analysis

#### 3.2.1 UV-visible and PL spectra

**Fig. 11:**
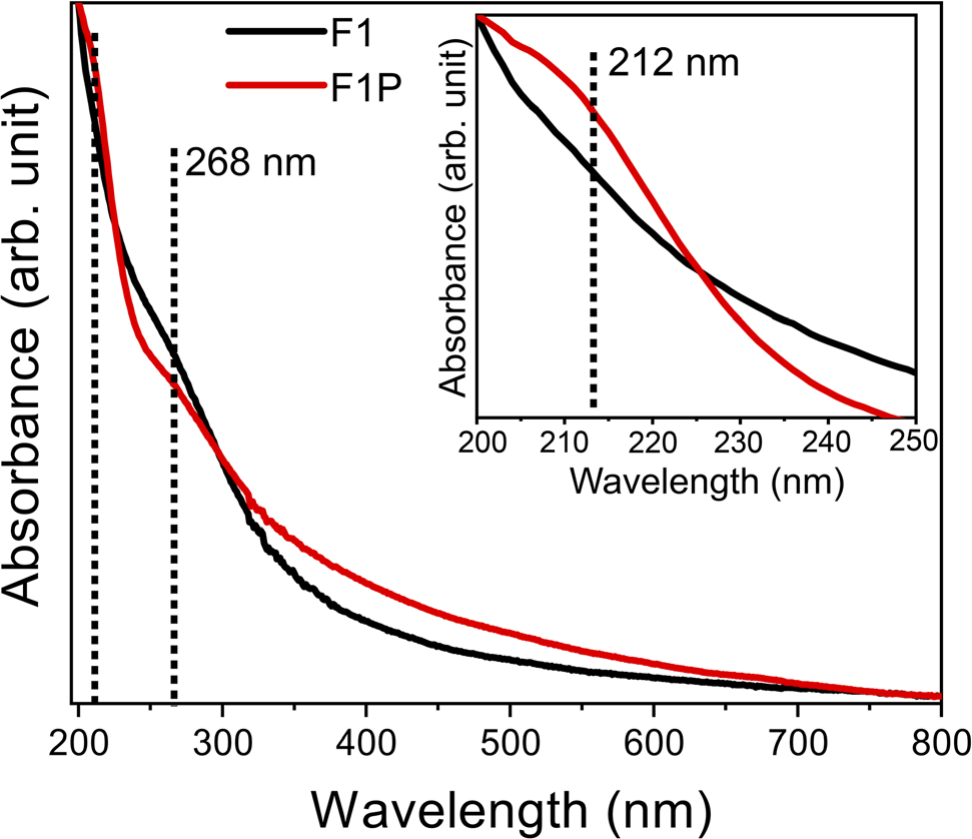
Absorbance spectra of F1 (black) and F1P (red), and in the inset the zoomed region of 200–250 nm is shown.

**Fig. 12:**
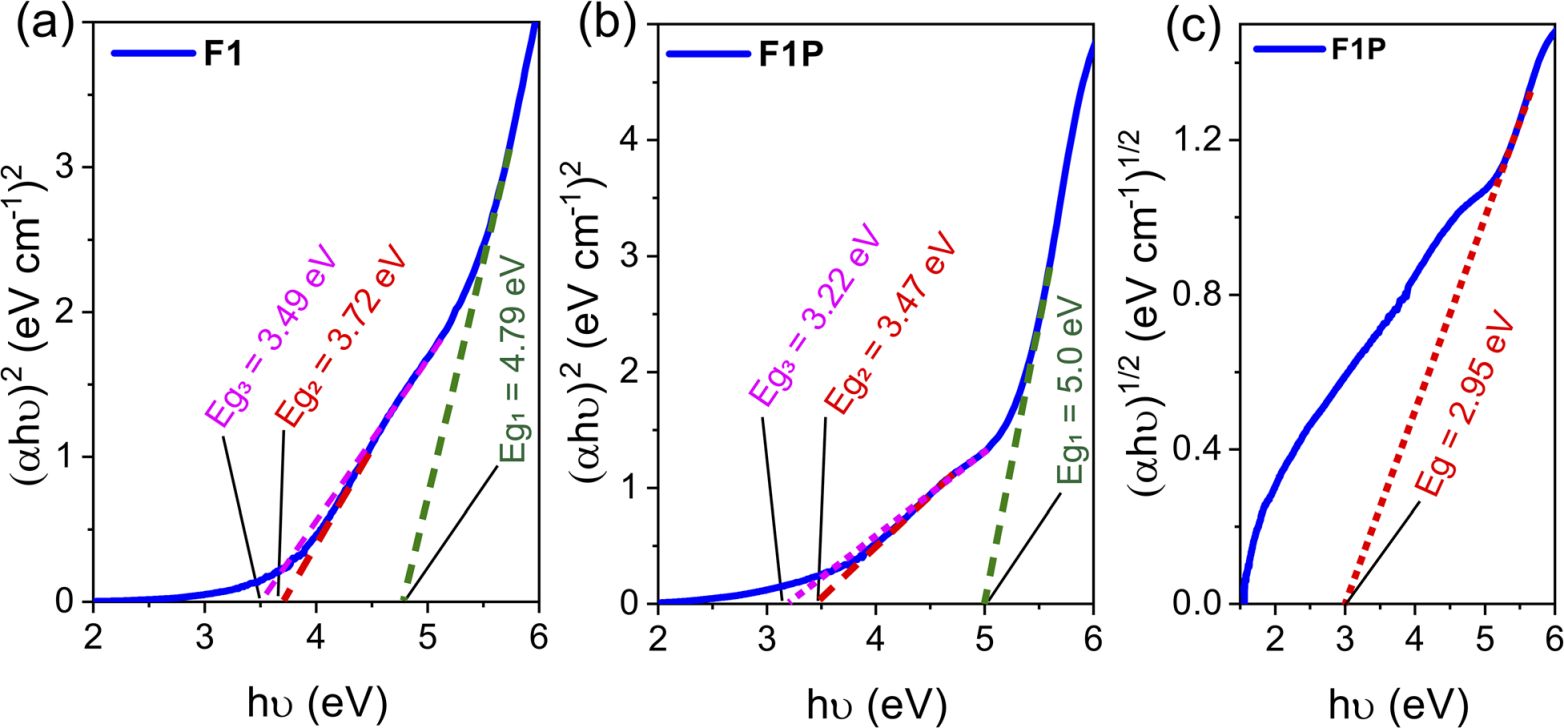
Tauc plot for (a) direct band transition in F1, (b) direct band transition in F1P; and (c) indirect band transition in F1P.

The absorbance spectra of both the samples (F1 and F1P) were obtained, as shown in Fig. 11. In case of F1, an absorbance peak at 268 nm was observed, which is attributed to Al and Fe present on the surface of the NPs getting oxidized and forming Al_2_O_3_ and Fe_3_O_4_.^2,37,76,77^ In F1P, in addition to the peak at 268 nm, another peak, although its weak, was observed around 212 nm (shown in the inset of Fig. 11), which is associated with the formation of Al_2_O_3_.^2,37,76^Fig. 12 shows the Tauc's plots derived from the absorbance spectra of both samples. From the Tauc plot, four band edges at 4.79 eV, 3.72 eV, 3.49 eV, and 3.38 eV, were noted. The first band edge (4.79 eV) is related to the bandgap between the conduction and valence band. This is lower than the reported bandgap for Al_2_O_3_ (5.3 eV).^76,78^ The lower bandgap, indicates the formation of Fe-doped Al_2_O_3_, where increase in the concentration of Fe leads to reduction in the bandgap. This is due to the sp–d interaction between Al and Fe, as well as substitution of Fe in place of Al, causing lattice distortion and introduces new electronic states within the band structure of Al_2_O_3_.^79^ The other band edges obtained from the Tauc plot are attributed to the presence of defect states originating from the oxidation of other elements present on the surface of the NPs.^80,81^

**Fig. 13:**
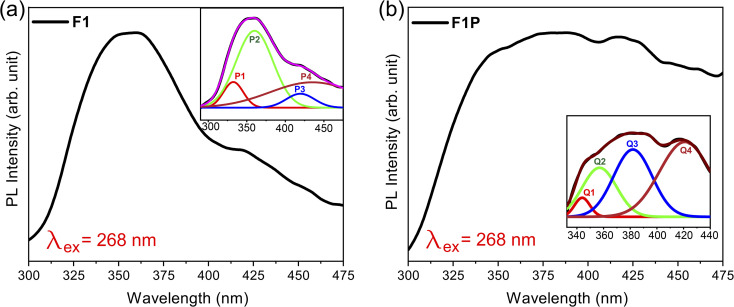
PL spectra of (a) F1 (with deconvoluted peaks shown in the inset) and (b) F1P NPs (with deconvoluted peaks shown in the inset).

**Fig. 14:**
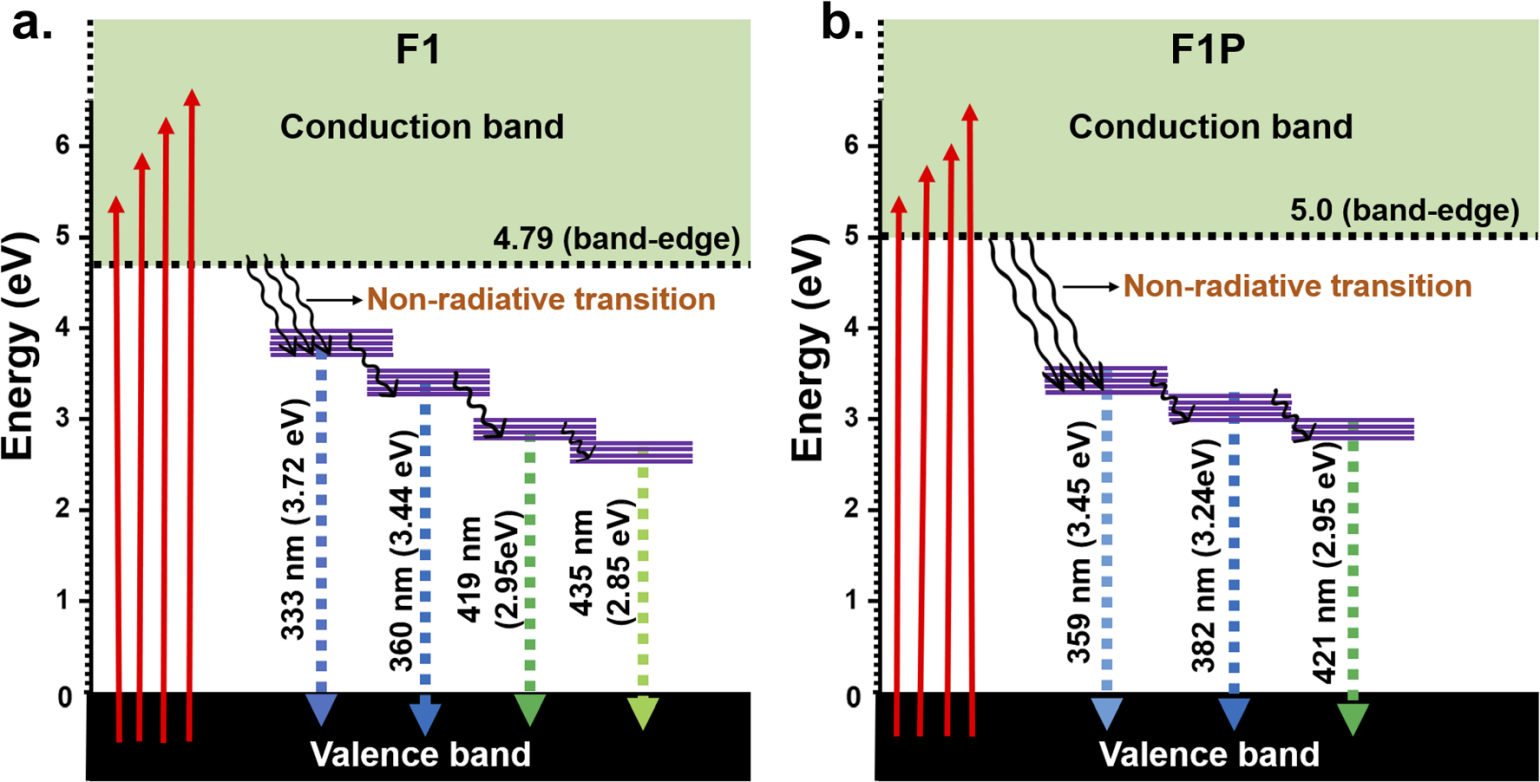
Energy level diagram for (a) F1 and (b) F1P (the defects states are shown with purple lines).

Similarly, for F1P, the bandgap was determined to be 5.0 eV, which is higher than the bandgap obtained for F1 and closer to the reported value of 5.3 eV for Al_2_O_3_. This can be due to the formation of Fe_5_Si_3_ phases after laser processing as mentioned earlier, which reduces/stops the formation of Fe-doped Al_2_O_3_. In addition, two other band edges, identified as defect states are observed at 3.22 eV and 3.47 eV. In both cases the band edge of 3.49 eV and 3.47 eV (for F1 and F1P) is attributed to the formation of defect states due to the oxidation of Fe, and forming Fe_3_O_4_.^77^ In contrast to F1, a indirect band edge was also detected for F1P sample (shown in Fig. 12(c)). To check the defect states identified from the Tauc plot, the PL spectra was monitored at an excitation wavelength of 268 nm. The PL spectra for the two samples is shown in Fig. 13. A broad emission was observed for F1 (Fig. 13(a)), and deconvolation of this broad peak (shown in the inset of Fig. 13(a)) revealed four peaks centered at 333 nm, 360 nm, 419 nm, and 435 nm, corresponding to emission energy of 3.72 eV, 3.44 eV, 2.95 eV and 2.85 eV respectively. The emission detected at 3.72 eV and 3.44 eV are in agreement with the band edges observed in the Tauc plot (Fig. 12(a)). On the other hand, in case of F1P, the PL spectra (Fig. 13(b)) showed a broader emission and its deconvolution (Fig. 13(b) inset) revealed, three strong emission peaks and two weak emission peaks. The strong emissions at 359 nm (3.45 eV) and 382 nm (3.24 eV), are in line with the band edges detected from the Tauc plot (Fig. 12(b)). In addition to this another strong peak at 421 nm (2.95 eV), and two weaker peaks at 344 nm (3.44 eV) and 394 nm (3.1 eV) are observed. The peak at 421 nm is identified as the indirect band transition (Fig. 12(c)). The weaker emission peaks (419 nm and 435 nm for F1; 344 nm and 394 nm for F1P) indicate the presence of additional defect states within the bandgap. Fig. 14 shows a schematic for the energy level diagram for the two samples.

## 4 Conclusion

In this study, FeMnNiAlSiC HEA NPs using a picosecond laser operating in burst-mode *via* PLAL was successfully synthesized. The structural analysis revealed B2-type, γ-brass, Fe_5_Si_3_-type, and BCC phases, as confirmed by HRTEM, SAED, and XRD. The broad peaks in the XRD pattern indicate that the NPs have a high degree of lattice strain and a small crystalline domain size, consistent with an amorphous/poor crystalline state. The observation of Fe_5_Si_3_-type phases during the burst-mode laser ablation pointed to the laser's inherent annealing effect. Elemental mapping revealed a compositional dependence of phase distribution: larger NPs were enriched with Al, Ni, and Fe, favorable for B2-type phase formation. While smaller NPs exhibited more Si and Mn, supporting the presence of Fe_5_Si_3_-type and γ-brass type phases. These structural insights were further validated by thermodynamic parameter calculations.

Further, the ablated NPs were subjected to LPL using a nanosecond laser. This secondary laser treatment resulted in significant morphological changes, such as the emergence of hollow HEA NPs alongside the previously observed solid spherical and core–shell structures. This transformation is attributed to asymmetric Ostwald ripening. The evolution of B2 and Fe_5_Si_3_-type phases in the processed NPs underscored the laser's annealing capability, mirroring the effects typically achieved through conventional furnace annealing. The subsequent nanosecond laser processing allows for more extensive heating and a slower cooling rate compared to picosecond pulses. This larger interaction promotes more effective thermal energy transfer to the NPs. The nanosecond laser processing acts as an annealing step. The prolonged heating period allows atoms more time to migrate/diffuse and rearrange into a more thermodynamically stable and ordered crystalline structure. The annealing effect reduces lattice strain and distortion by enabling relaxation of the crystal lattice. This process helps in reducing defects and improving the overall crystallinity of the NPs. The sharper peaks in the XRD pattern after processing indicate an improved crystallinity. The narrow peaks suggest larger crystalline domains and reduced strain, reflecting a transition from an amorphous/poor crystalline state to a well-ordered crystalline structure.

These findings highlight the potential of pulsed laser techniques for tailoring the structural and morphological properties of HEA NPs. This dual-stage laser processing approach offers a versatile and efficient method for enhancing NPs' properties. In the present study, we have used a fixed burst-mode setting with 10 pulses per burst, which may limit the understanding of how burst parameters influence NPs formation. In future work, the number of pulses per burst will be systematically varied to investigate its effect on particle size, phase formation, and crystallinity, enabling a deeper understanding of burst-mode laser ablation for tailored HEA nanoparticle synthesis, paving the way for their application in advanced functional materials.

## Author contributions

Bibek Kumar Singh: investigation, writing – original draft, Yagnesh Shadangi: writing – review and editing. Harsh Jain: data curation. R. Sai Prasad Goud: data curation. N. K. Mukhopadhyay: writing – review and editing. A. P. Pathak: writing – review and editing. S. Venugopal Rao: writing – review and editing. Archana Tiwari: conceptualization, supervision, writing – review and editing. Ajay Tripathi: conceptualization, supervision, writing – review and editing.

## Conflicts of interest

There are no conflicts to declare.

## Supplementary Material

RA-015-D5RA03923A-s001

## Data Availability

The data supporting this study's findings are available from the corresponding author upon reasonable request, and some of the data are made available in the SI file. The SI includes additional characterization data supporting the main findings, such as HRTEM, SAED, and particle size distribution of F1P nanoparticles, Raman spectra of F1 and F1P, elemental mapping and line-scan analyses, EDS spectra with recalculated compositions, and XRD patterns of the target before and after annealing. It also provides thermodynamic and physico-chemical parameters derived from corrected elemental data. See DOI: https://doi.org/10.1039/d5ra03923a.
