# Supplementary material for: Phase evolution and morphological transformation of high-entropy alloy FeMnNiAlSiC nanoparticles via sequential picosecond laser ablation and nanosecond laser annealing
Source: RSC Adv. 2025 Aug 11;15(35):28395–412. doi: 10.1039/d5ra03923a (PMC12376931; doi:10.1039/d5ra03923a)
Supplement: RA-015-D5RA03923A-s001 [file RA-015-D5RA03923A-s001.pdf]

Supplementary Information (SI) for RSC Advances.
This journal is © The Royal Society of Chemistry 2025

Journal Name

ARTICLE TYPE

Cite this: DOI: 00.0000/xxxxxxxxxx

Phase Evolution and Morphological Transformation of High-Entropy Alloy Nanoparticles via Sequential Picosecond Laser Ablation and Nanosecond Laser Annealing†

Bibek Kumar Singh,[a] Yagnesh Shadangi,[b] Harsh Jain,[c] R. Sai Prasad Goud,[d,e] N. K. Mukhopadhyay,[f] A. P. Pathak,[g] Venugopal Rao Soma,[g,h] Archana Tiwari,[i] and A. Tripathi*[a]

Fig. S1(a) shows the TEM image of the F1P NPs, showing the smaller NPs with its particle size distribution shown in the inset. The avergae size of the small-size NPs was calculated as 17 nm (± 2 nm). Fig. S1(b) shows the HRTEM of a F1P NP. From this, B2-type phase (d-spacing of 0.236 nm, 0.232 nm, and 0.227 nm corresponding to (330), (-332), and (512) planes, respectively), γ-brass type phase (d-spacing of 0.256 nm corresponding to (222) plane), and Fe_5Si_3-type phase (d-spacing of 0.217 nm corresponding to (210) plane) were identified [JCPDS No.: 00-029-0042, 01-083-3994; 00-006-0696, 00-001-1262; 01-071-0397; 00-011-0615,01-074-4749; 00-001-0787, 00-027-1402]. Fig. S1(c), shows the SAED pattern obtained from the F1P NPs. From the SAED pattern, d-spacing values corresponding to B2-type phase (0.281 nm and 0.242 nm, corresponding to (-512) and (024) planes, respectively), BCC phase (0.117 nm, coressponding to (211) plane), γ-brass-type phase (0.137 nm, corresponding to (541) plane), and Fe_5Si_3-type phase (0.234 nm and 0.165 nm, corresponding to (002) and (220) planes, respectively) [JCPDS No.: 00-029-0042, 01-083-3994; 00-006-0696, 00-001-1262; 01-071-0397; 00-011-0615,01-074-4749; 00-001-0787, 00-027-1402].

Fig. S2 and S3 shows the Raman spectra of F1 and F1P NPs respectively. The peaks observed here are all associated with Si[1]. In both the cases peaks related to C was not observed, indicating that the C concentration has not been significantly affected due to laser ablation and processing. The elemental mapping of F1 NPs is shown in Fig. S4(a). In addition, two more particles marked as 3 and 4 in Fig. S4(a), were checked separately and their elemental mapping is shown in Fig. S4(b) and (c), respectively. The mapping image obtained for carbon is shown separately in Fig. S5. Further, the line scan of F1 and F1P NPs, is displayed in Fig. S6 and S7, respectively. The distribution of the NPs observed from the line scan in bith the cases are found to in agreement with there elemental distribution as discussed in the main text file. The EDS of F1 and F1P is shown in Fig. S8 and S9, respectively. Due to the use of Carbon based substrate, the concentration of C was found to be highest (see Fig. S5) in both cases. In order to calculate the physio-chemical parameters and thermodynamic parameters (atomic radius, self diffusion coefficient, atomic size deviation (δ), valence electron concentration (VEC), Gibbs free energy (ΔG_{mix}), entropy of mixing (ΔS_{mix}), enthalpy of mixing (ΔH_{mix}), melting temperature(T_m)), the elemental composition was re-evaluated after fixing the at.% of C at 1%.

[a] Department of Physics, School of Physical Sciences, Sikkim University 6th mile Samdur, 737102, Sikkim, India; E-mail: ajay_t_2000@yahoo.com
[b] Department of Materials Science and Metallurgical Engineering, Indian Institute of Technology, Bhilai, Jevra-Sirsa Road, Durg, Chhattisgarh, 491001, India.
[c] Department of Ceramic Engineering, Indian Institute of Technology (BHU), VaranasiâĂŞ 221005, Uttar Pradesh, India
[d] Centre for Advanced Studies in Electronics Science and Technology (CASEST), School of Physics, University of Hyderabad, Hyderabad, 500046, Telangana, India
[e] Centre for Nanotechnology, University of Hyderabad, 500046, Telangana, India
[f] Department of Metallurgical Engineering, Indian Institute of Technology (BHU), VaranasiâĂŞ 221005, Uttar Pradesh, India
[g] School of Physics, University of Hyderabad, Hyderabaad, 500046, Telangana, India
[h] DRDO Industry Academia - Centre of Excellence (DIA-CoE;formerly ACRHEM), University of Hyderabad, Hyderabad500046 Telangana, India
[i] Department of Physics, Institute of Science, Banaras Hindu University, Varanasi, 221005, Uttar Pradesh, India

† Supplementary Information available: [details of any supplementary information available should be included here]. See DOI: 00.0000/00000000.

Recalculation of Elemental Percentages after Fixing Carbon Content

In the original EDS data, the atomic percentages of all elements summed to 100%. However, since the sample was analyzed on a carbon-based grid, the detected carbon content was found to be elevated. To account for this, the carbon content was fixed at **1 at.%**, and the percentages of the remaining elements were renormalized to sum to 99 at.% using the following relation:

$$C_i^{\text{new}} = C_i^{\text{orig}} \times \frac{99}{\sum C_j^{\text{orig}} - C_{\text{C}}^{\text{orig}}} \quad (1)$$

where, C_i^{orig} is the original atomic percentage of element i, $C_{\text{C}}^{\text{orig}}$ is the original atomic percentage of carbon, and C_i^{new} is the recalculated atomic percentage.

The uncertainty (standard deviation, σ)) for each recalculated percentage was also calculated using the following equatons:

$$\sigma_i^{\text{new}} = \sigma_i^{\text{orig}} \times \frac{C_i^{\text{new}}}{C_i^{\text{orig}}} \quad (2)$$

For the fixed carbon content (1 at.%), the associated uncertainty was scaled according to:

$$\sigma_{\mathrm{C}}^{\mathrm{new}} = \sigma_{\mathrm{C}}^{\mathrm{orig}} \times \frac{1}{C_{\mathrm{C}}^{\mathrm{orig}}} \tag{3}$$

The physio-chemical parameters and thermodynamic parameters of alloys determined using Eqs. 1 - 7[2–5], are shown in Table T1 and T2 for F1 and F1P, respectively.

$$\Delta G_{mix} = \Delta H_{mix} - T\Delta S_{mix} \tag{4}$$

$$\Delta S_{mix} = -R\sum_{i=1}^{N}(C_i lnC_i) \tag{5}$$

$$\Delta H_{mix} = \sum_{i=1,i\neq j}^{N}(4\Delta H_{mix}^{AB}C_iC_j \tag{6}$$

$$\Omega = |\frac{T_m\Delta S_{mix}}{\Delta H_{mix}}| \tag{7}$$

$$\delta = \sqrt{\sum_{i=1}^{N}C_i(1-\frac{r_i}{r})^2} \tag{8}$$

$$\bar{r} = \sum_{i=1}^{N}C_i r_i \tag{9}$$

$$VEC = \sum_{i=1}^{N}C_i(VEC)_i \tag{10}$$

The XRD pattern for the bulk target, before annealing, and after annealing is shown in Fig. S10, and S11 respectively.

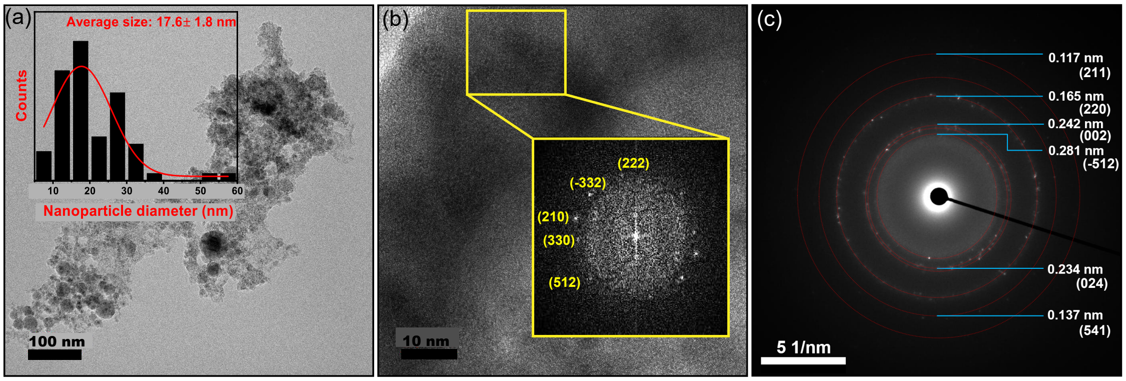

Fig. S1 (a) TEM image with its particle size distribution shown in the inset, (b) HRTEM image with its FFT shown in the inset (the yellow box marks the region from where the FFT was obtained), (c) SAED pattern, of F1P NPs.

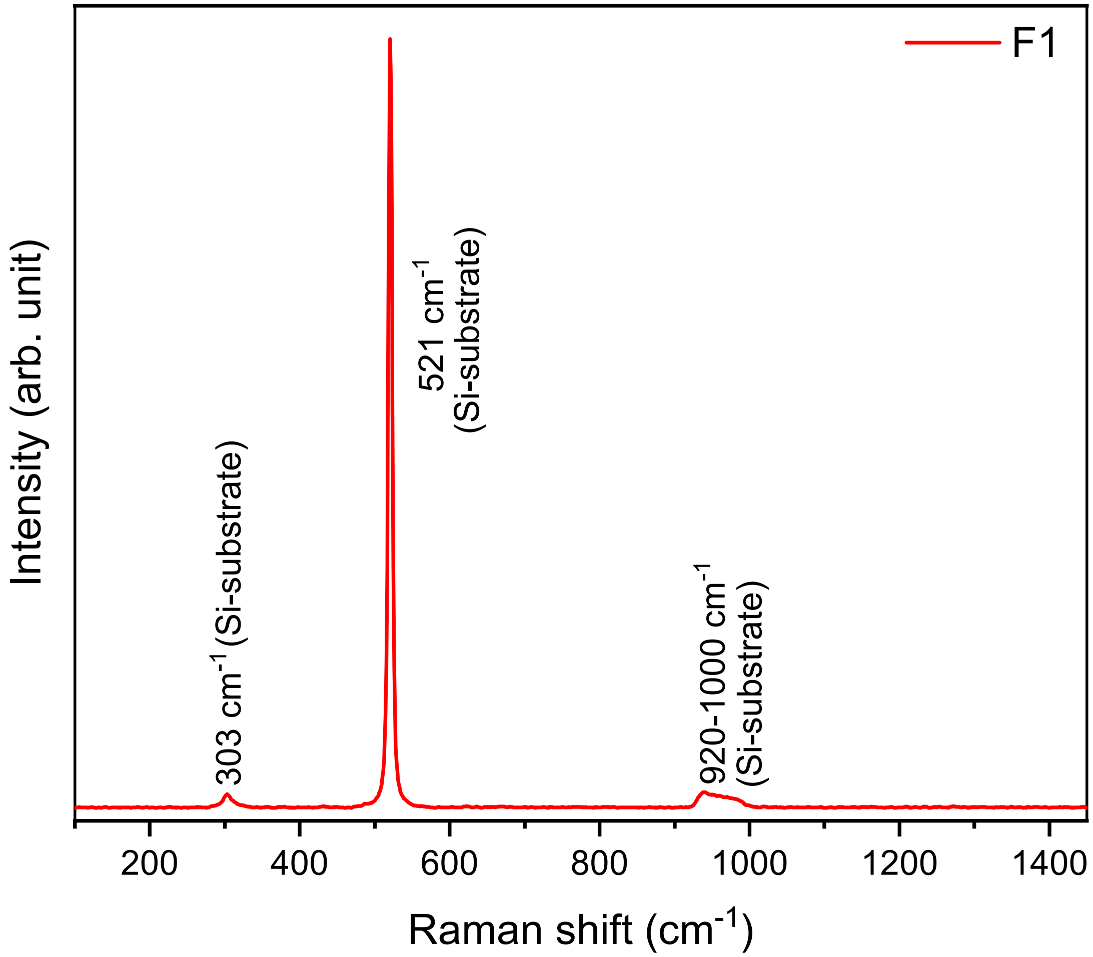

Fig. S2 Raman spectra of F1 NPs.

Table T1 Thermodynamic parameters of F1 NPs

Region	ΔS_{mix} (J/mol.K)	ΔH_{mix} (kJ/mol)	$T_m(K)$	δ (%)	Ω	VEC
1	12.32	-37.27	1667.4	7.91	0.55	6.24
2	12.36	-31.79	1662.1	7.68	0.65	6.56

Table T2 Thermodynamic parameters of F1P NPs

Region	ΔS_{mix} (J/mol.K)	ΔH_{mix} (kJ/mol)	$T_m(K)$	δ (%)	Ω	VEC
1	10.43	-15.60	1653.7	7.14	1.1	7.05
2	12.32	-30.53	1644.2	7.91	0.66	6.49

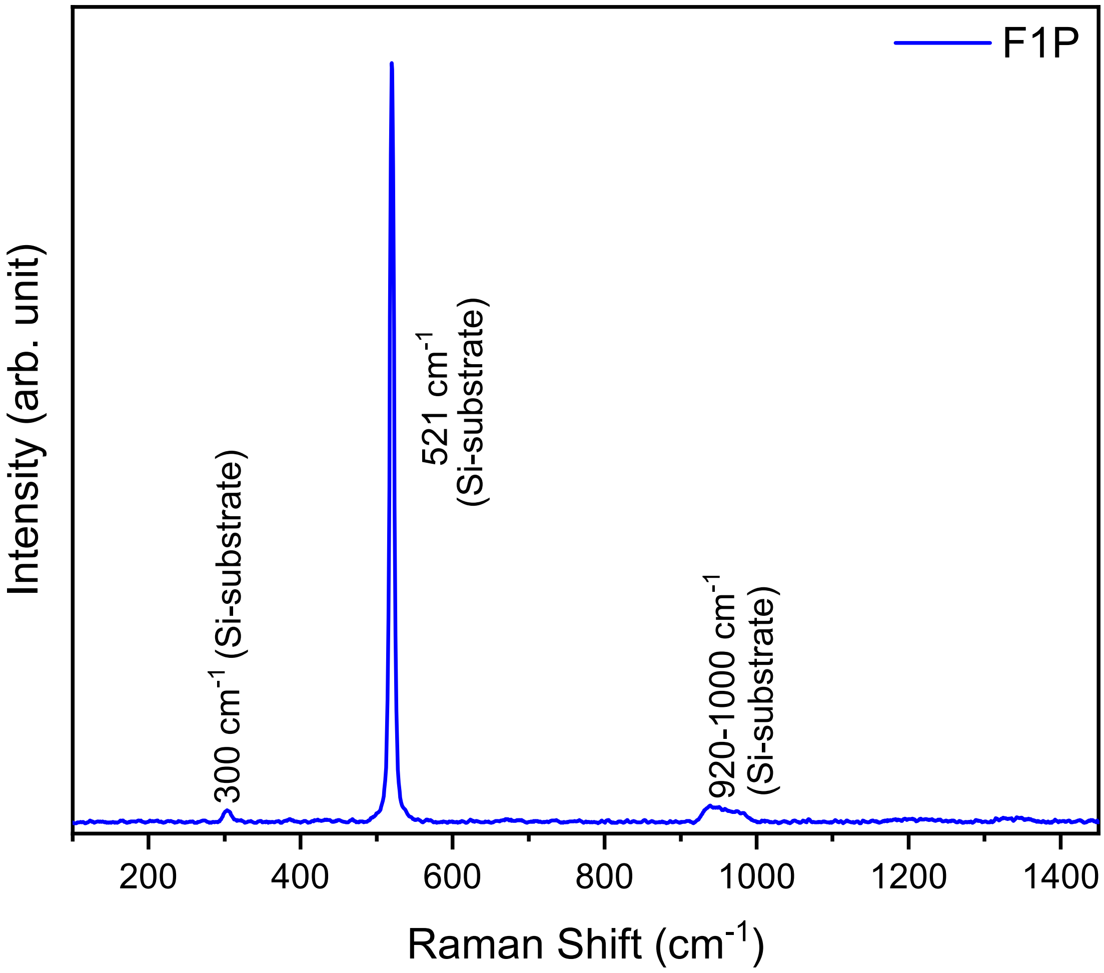

Fig. S3 Raman spectra of F1P NPs.

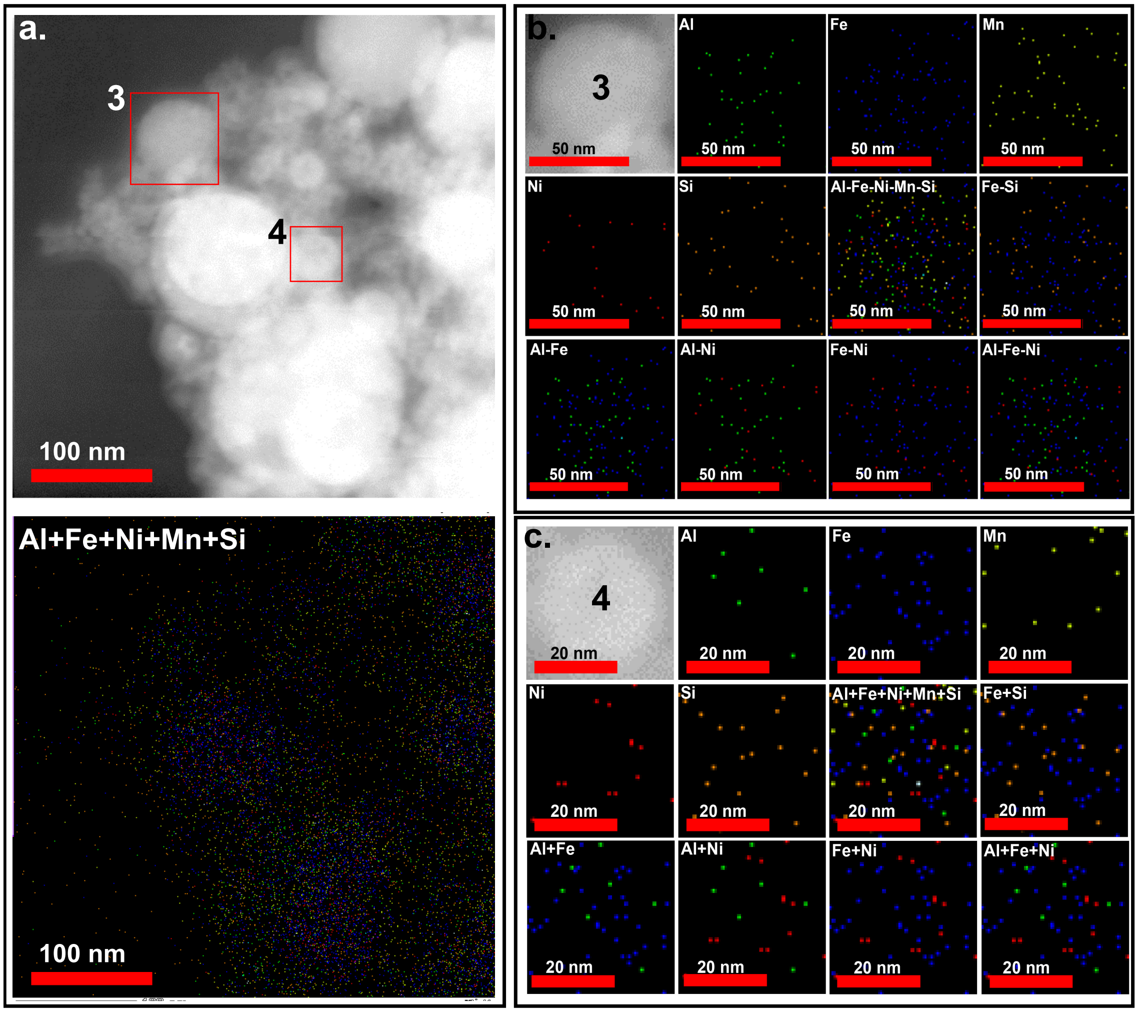

Fig. S4 (a) SEM image and its corresponding elemental mapping, (b-c) Elemental mapping of particle 3 and 4 marked in (a), respectively, of F1 NPs.

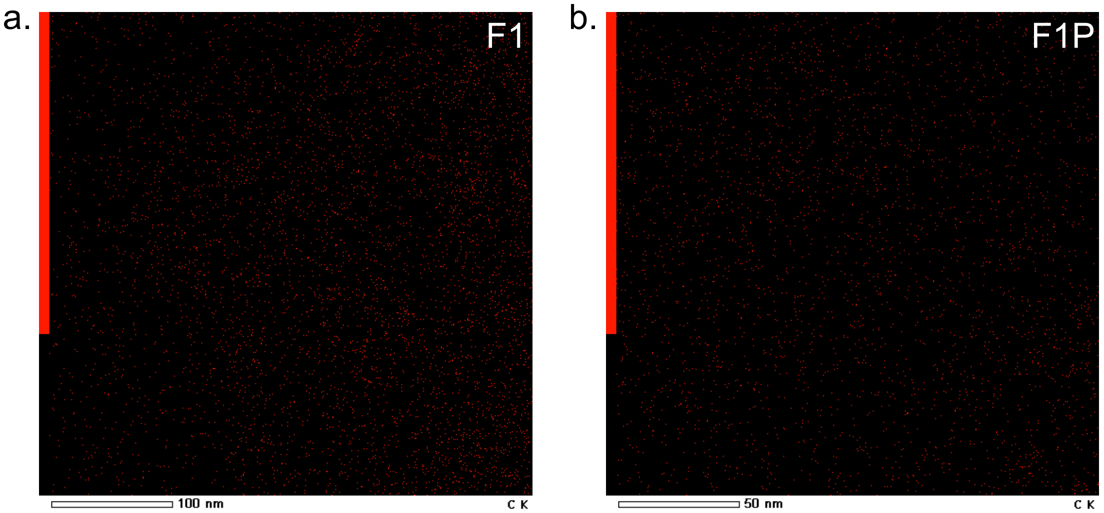

Fig. S5 Elemental mapping obtained C for (a) F1 NPs, and (b) F1P NPs, respectively

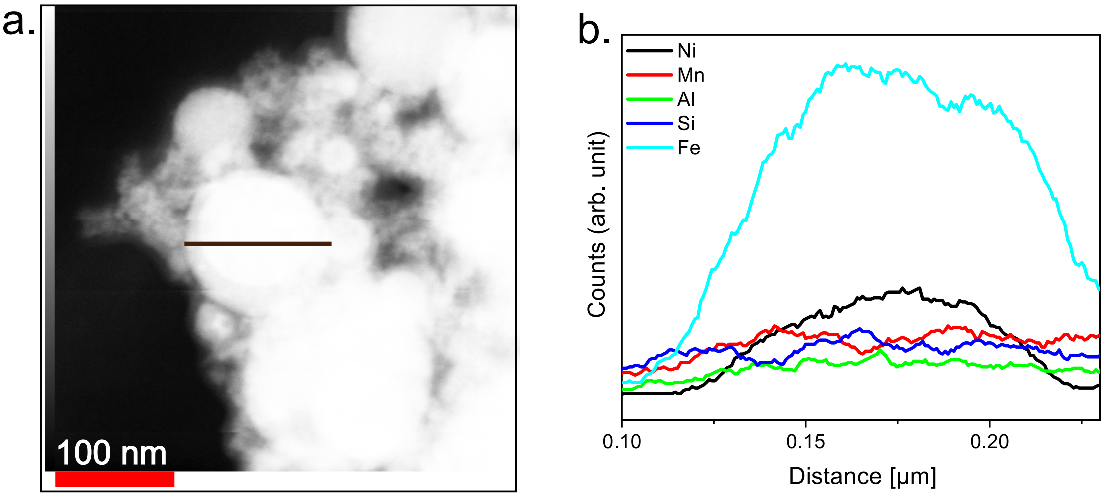

Fig. S6 (a) SEM image of F1 NP, and (b) Line-scan obtained from region marked in (a).

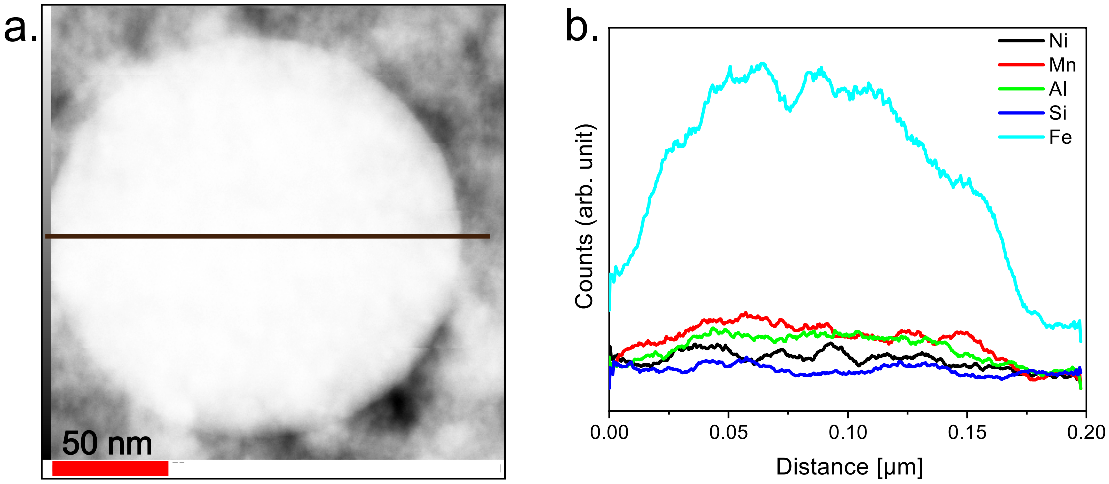

Fig. S7 (a) SEM image of F1P NPs, and (b) Line-scan obtained from region marked in (a).

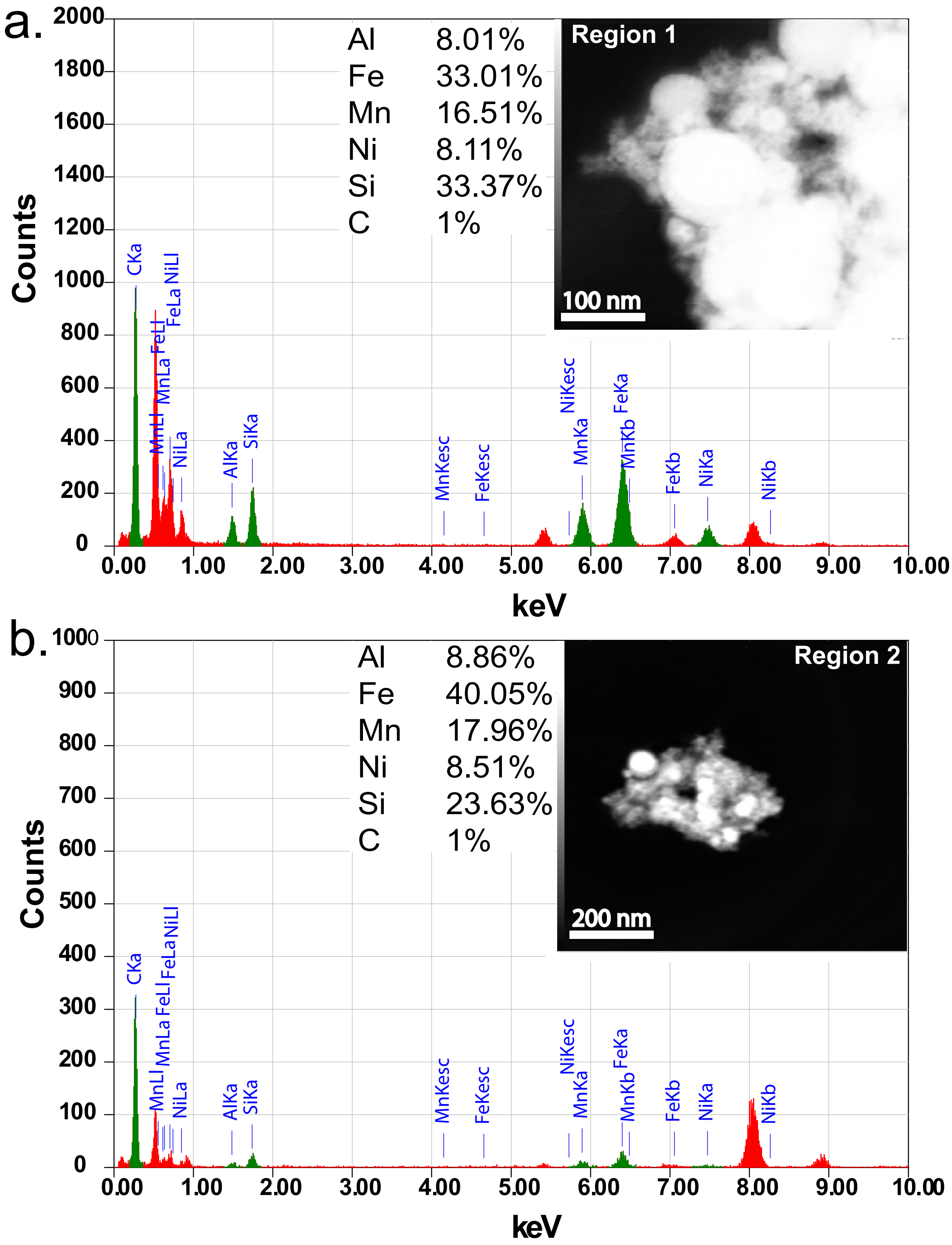

Fig. S8 (a-b) EDS of F1 obtained from two different regions, in the inset the corresponding region is shown along with the recalculated at.% of the constituent elements.

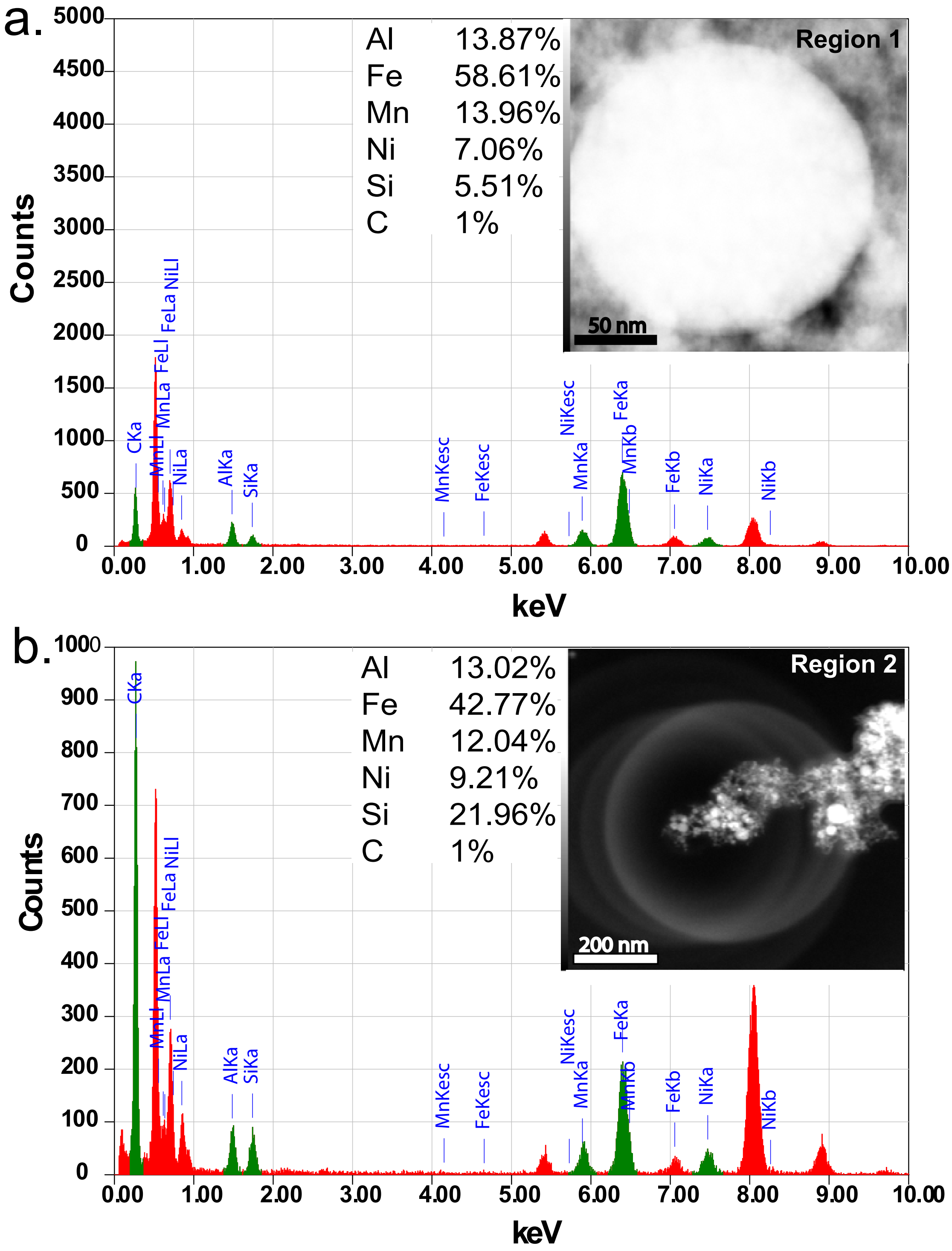

Fig. S9 (a-b) EDS of F1P obtained from two different regions, in the inset the corresponding region is shown along with the recalculated at.% of the constituent elements.

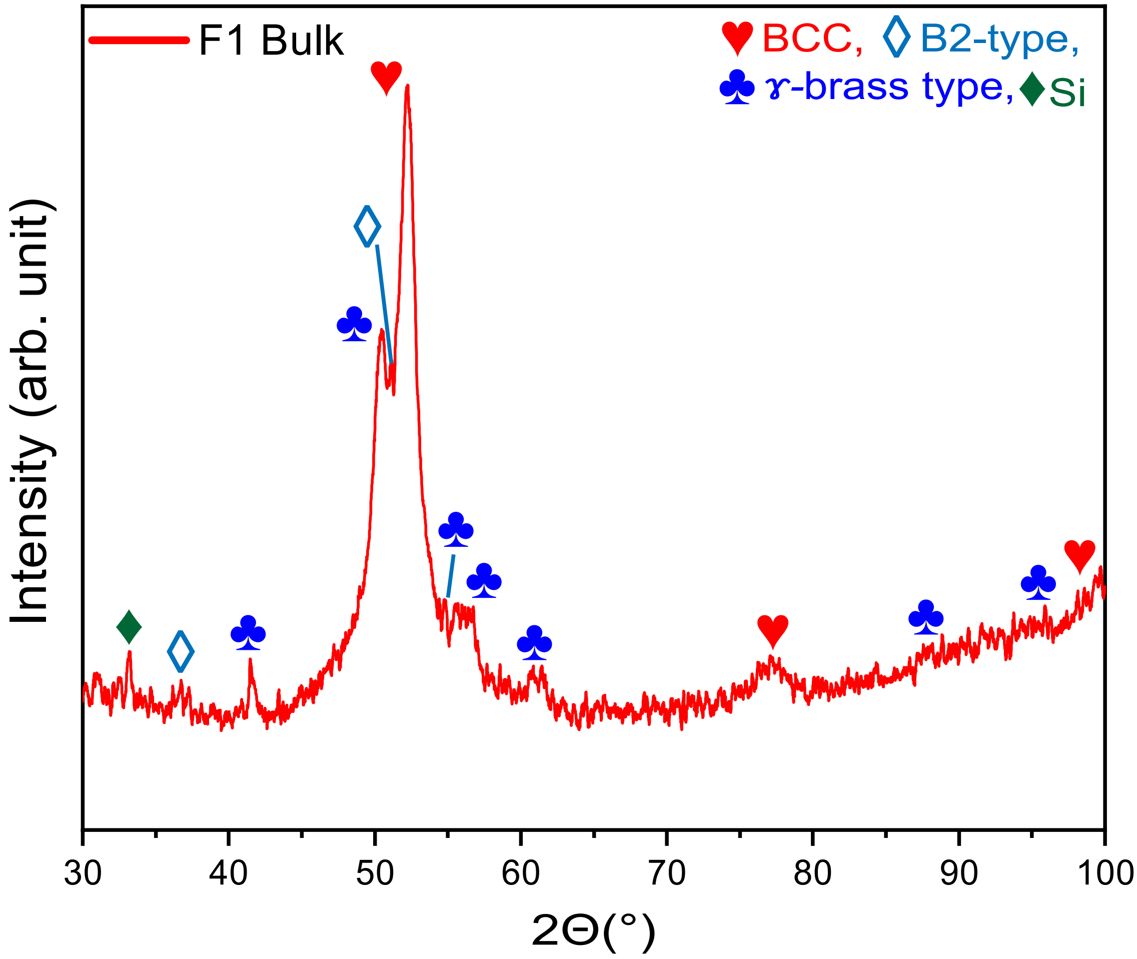

Fig. S10 XRD of target.

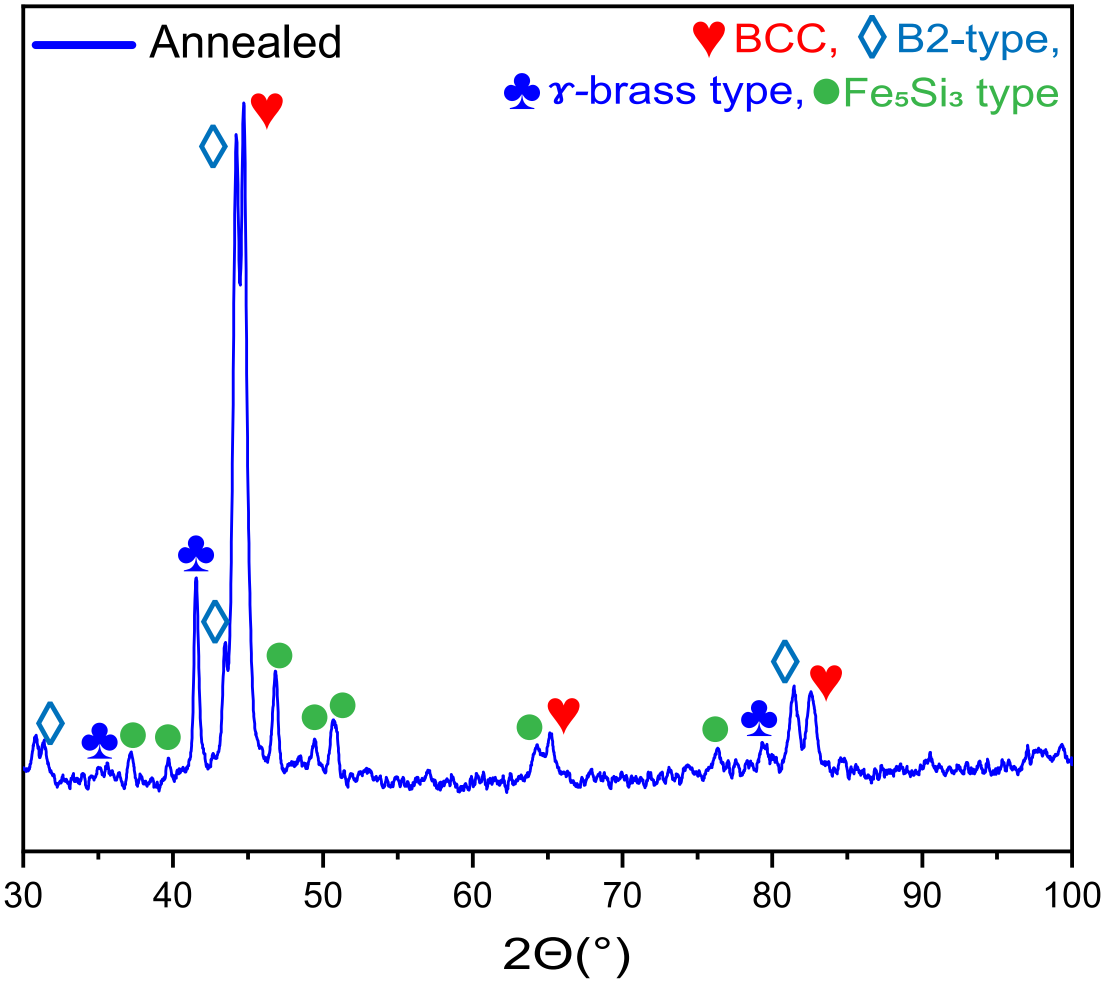

Fig. S11 XRD of target after annealing (800°C).

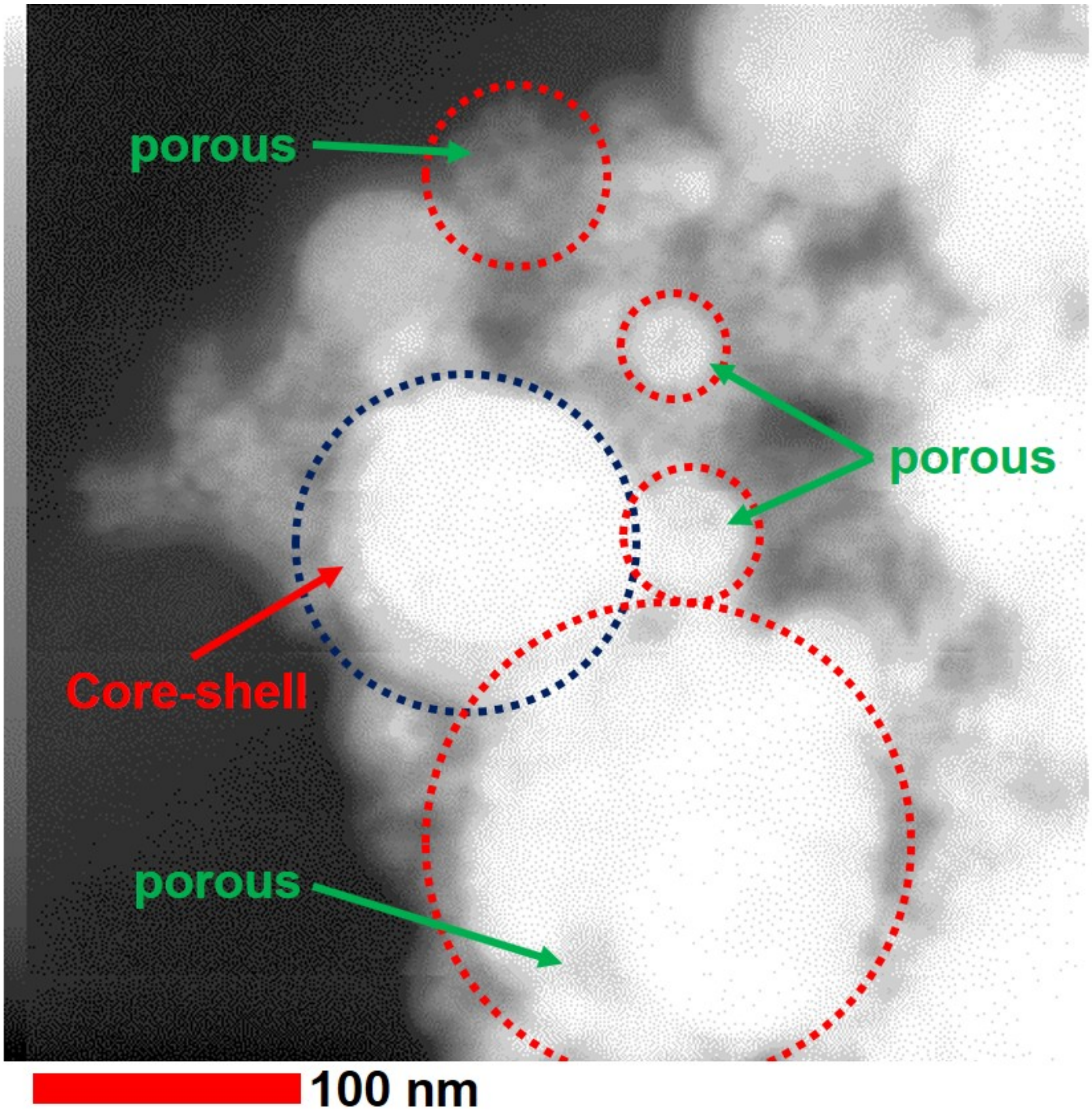

Fig. S12 SEM image of F1 NPs, highlighting the porous and core-shell structures.

References

1 W.-J. Lee and Y.-H. Chang, *Coatings*, 2018, **8**, 431.

2 Y. Zhang, Y. J. Zhou, J. P. Lin, G. L. Chen and P. K. Liaw, *Advanced engineering materials*, 2008, **10**, 534–538.

3 G. Sheng and C. T. Liu, *Progress in Natural Science: Materials International*, 2011, **21**, 433–446.

4 X. Yang and Y. Zhang, *Materials Chemistry and Physics*, 2012, **132**, 233–238.

5 R. Rawat, B. K. Singh, A. Tiwari, N. Arun, A. P. Pathak, Y. Shadangi, N. Mukhopadhyay, S. R. Nelamarri, S. V. Rao and A. Tripathi, *Journal of Alloys and Compounds*, 2022, **927**, 166905.
